# A repository of Singapore validated PROMS: a scoping review

**DOI:** 10.1186/s41687-026-01005-4

**Published:** 2026-02-23

**Authors:** Yu Heng Kwan, Christy Lim, Esther Ja Hooi Chew, Ming Feng Gabriel Gan, Charmaine Wai Yan Sum, Xin Ru Chew, Hairil Rizal Abdullah, Charles Goh, Jordan Bai, Meixiang Winnie Koh, Ellie Choi, Lian Leng Low, Warren Fong, Ying Ying Leung, Sungwon Yoon, Silvana Choo, Julian Thumboo

**Affiliations:** 1 Department of Rheumatology and Immunology, Singapore General Hospital, The Academia, Level 4, 20 College Road, Singapore, 169856 Singapore; 2 Centre of Population Health and Implementation Research, SingHealth Regional Health System, Singapore, Singapore; 3 Department of Anesthesiology, Singapore General Hospital, Singapore, Singapore; 4 Department of Nuclear Medicine and Molecular Imaging, Singapore General Hospital, Singapore, Singapore; 5 Value, Safety & Performance Division, Ministry of Health, Singapore, Singapore; 6 Department of Clinical & Academic Development, National Healthcare Group, Singapore, Singapore; 7 Division of Dermatology, Department of Medicine, National University Hospital, Singapore, Singapore; 8 Post Acute and Continuing Care, Outram Community Hospital, Singapore, Singapore; 9 Department of Occupational Therapy, Singapore General Hospital, Singapore, Singapore

**Keywords:** Scoping review, Reliability and validity, Patient-reported outcome measures, Value driven care, Repository

## Abstract

**Purpose:**

We aimed to conduct a scoping review to create a repository of validated Patient-reported outcome measures (PROMs) in Singapore, listing relevant psychometric properties assessed and identifying if these PROMs are assessed for the Value Driven Care (VDC) conditions in Singapore.

**Methods:**

This study was guided by the Preferred Reporting Items for Systematic Reviews and Meta-Analyses Extension for Scoping Reviews guidelines. Relevant articles were retrieved from PubMed^®^, Embase^®^, Scopus^®^ and PsycINFO^®^ with additional searches from Factiva^®^, and Proquest^®^ databases published up to 19 September 2024. Selected PROMs were categorised based on the COnsensus-based Standards for the selection of health Measurement Instruments guidelines with psychometric properties into 4 main categories, including reliability (internal consistency and measurement error), validity (content validity, criterion validity, and construct validity), responsiveness, and interpretability. We also specified if these PROMs were validated in Singapore in patients with VDC conditions.

**Results:**

A total of 115 locally validated PROMs from 165 studies were identified, with EuroQol 5 Dimension questionnaire and 36-Item Short Form Health Survey having the greatest number of studies. These PROMs were used across 35 different conditions, with musculoskeletal diseases being the most common area for locally validated PROMs while diseases like pneumonia, caesarean section, and hemorrhoidectomy, lack validated PROMs. Validity and reliability were the most frequently assessed measurement properties.

**Conclusion:**

This scoping review established a repository of 115 PROMs validated for use in Singapore. This repository can guide clinicians and researchers in PROMS selection and in identifying conditions where further PROMs validation studies are needed to improve care.

**Supplementary Information:**

The online version contains supplementary material available at 10.1186/s41687-026-01005-4.

## Introduction

Patient reported outcome (PROMs) are questionnaires that provide the patients' perspectives on their health status and quality of life over time [1], and can also be one of the key indicators to capture outcomes from the patient’s perspective [2]. There are two broad types of PROMs — generic and disease-specific respectively and they are extensively employed for different purposes across the micro, meso, and macro levels [3]. 

At the micro level, PROMs enhance clinician-patient interactions and compare the effects of different treatments to understand variations among healthcare providers related to the meso level. Subsequently, it can be beneficial for population surveillance and informing national healthcare-related policies at the macro level [4]. However, there are many logistical, social, cultural, technical and legal barriers to implementing suitable PROMs that can be applied widely within our healthcare practice [1]. Varying definitions and terminologies currently exist in the field of practice with varied approaches in PROMs methodologies [1]. Hence, the integration of PROMs in routine clinical care is dismal due to insufficient standardisation and robust translation of PROMs made available within the local context [1]. 

Singapore has restructured its public healthcare system, into three healthcare clusters publicly funded by Singapore’s Ministry of Health [5]. Each healthcare cluster is charged with the health of one region of Singapore for example Singapore Health Services (SingHealth) for the eastern of Singapore, National Healthcare Group for the central part of Singapore, and National University Health System for the western part of Singapore [5]. Increasingly in the public healthcare clusters there is a movement towards Value Driven Care (VDC) [6]. The VDC is a healthcare management model focused on meeting patient needs and achieving optimal health outcomes while ensuring cost efficiency through high-quality and cost-effective healthcare services, alongside effective management and utilisation of medical resources [7]. The value of care can be measured using PROMs to assess quality of health outcomes in relation to the healthcare costs incurred [8]. Presently, Singapore’s Ministry of Health (MOH) has identified 19 VDC conditions as “high impact” conditions from a national perspective and is a crucial initiative in supporting MOH’s ‘3 Beyonds’ — Beyond Healthcare to Health, Beyond Hospital to Community and Beyond Quality to Value [6, 9]. The conditions are chosen due to their prevalence with incidence expected to increase due to the aging population in Singapore [10]. Consequently, by focusing on VDC, Singapore could further improve patient experiences, offering personalised care that better meets individual needs and encourages positive health outcomes.

The development of a validated PROM repository for Singapore will enable more effective management of VDC conditions for several reasons, leading to better healthcare outcomes that align with patient expectations [11]. First, systematic collection of patient feedback strengthens communication between patients and healthcare providers, fostering a deeper understanding of patients’ needs and preferences, and ultimately enhancing patient satisfaction [12]. Second, PROMs raise patients’ awareness of their symptoms and overall health, empowering them to take a more active role in managing their conditions [13]. Disease-specific PROMs are designed to capture particular symptoms and their impact on the function of the condition, in this case, VDC conditions [11]. Third, a comprehensive PROM list facilitates the evaluation of healthcare performance across various local healthcare institutions, promoting competition and driving improvements in care quality for patients. The consolidated repository will be maintained by the SingHealth cluster. The repository will be available to the other healthcare clusters as needed.

Utilising PROMs for the management of VDC conditions will therefore ensure that healthcare delivery is patient-centred by making informed decisions, improving both the quality of care and patient outcomes [11]. The use of PROMs has been well established in clinical trials for many years [1]. Additionally, in recent years, PROMs have also been increasingly utilised in routine clinical care to facilitate shared decision-making between clinicians and patients [11]. 

Some online resources and databases, such as the Patient-Reported Outcome and Quality of Life Instruments Database (PROQOLID), provide detailed information on various PROMs instruments [14]. However, to date, there is no comprehensive repository or standardised list of PROMs readily accessible for clinicians to gather patient-reported outcomes tailored for local culture and use. This lack of a centralised resource further hinders the adoption and consistent use of PROMs in Singapore’s clinical practice. A central repository would provide researchers, clinicians and administrators, a consolidated reference point for PROMs. It will enable researchers to identify areas of need to focus efforts on, clinicians to select the appropriate outcomes to be validated, and administrators to reduce time required to plan an appropriate evaluation program. Therefore, implementing a suitable locally validated list of PROMs is crucial to strengthening the healthcare system by improving patient-centric care [1]. 

Hence, we aimed to perform a scoping review that aims to identify and summarise studies that validated PROMs in Singapore. We also listed the relevant psychometric properties assessed for and if these PROMs are listed under the MOH VDC conditions.

## Methodology

This scoping review is registered under the Open Science Framework (registration number A7HXU) and performed according to the Preferred Reporting Items for Systematic Reviews and Meta-Analyses Extension for Scoping Reviews (PRISMA-ScR) guidelines [15]. 

### Search strategy

This study used a three-pronged search strategy to identify relevant literature — querying literature databases, targeting top journals, and utilising snowball sampling. First, we conducted a search for articles published up to 19 September 2024 across PubMed^®^, Embase^®^, Scopus^®^, and PsycINFO^®^ databases using relevant keywords relating to Singapore, Patient-Reported Outcome Measures (PROMs), and various psychometric properties singly and in combination (full search strategy provided in Supplementary Table 1). To enhance search sensitivity, we incorporated search filters created by local researchers, which combined specific terms [16, 17]. For the PROMs construct, synonyms such as “self-reported” were also included in the search. Duplicates were removed from the final dataset. The extracted sources were then manually filtered based on their relevance to VDC conditions and their alternative medical terminology. Additionally, a grey literature search was performed to include other resources from Factiva^®^ and Proquest^®^ to capture other relevant resources. Advice from the Senior Medical Research Librarian specialising in science, technology, engineering and mathematics (STEM) disciplines at a university was sought to validate the above search strategy [18]. 

### Inclusion and exclusion criteria

Articles included in this study were full-text publications in English performed in Singapore, with the assessment of at least one measurement property listed in the COSMIN. We excluded studies that were not written in English, not performed in Singapore, systematic reviews or made no mention of PROMs psychometric properties. In total, 2 independent reviewers (CL, EC) screened the titles and abstracts of the studies according to the inclusion and exclusion criteria. Opinions from a third reviewer (GG) were sought in the event of any disagreements. The remaining full-text articles were then evaluated by the same 2 independent reviewers for inclusion and exclusion.

### Data extraction

Data were extracted from the included articles by two independent reviewers, when available. The collected data included the country of origin (Singapore) and a list of VDC conditions, such as total knee replacement, cataract surgery, laparoscopic cholecystectomy, hysterectomy, coronary artery bypass graft, hernia repair, caesarean section, tonsillectomy, haemorrhoidectomy, total hip replacement, acute myocardial infarction, congestive heart failure, ischemic stroke, pneumonia, colorectal resection, spinal fusion, breast cancer, end-of-life care, and diabetes mellitus. The review also included an analysis of PROM characteristics based on psychometric properties, such as reliability, internal consistency, measurement error, content validity, criterion validity, construct validity, responsiveness, and interpretability. Additionally, we extracted study characteristics, including gender, age, PROMs, psychometric properties, and their corresponding VDC conditions.

### Data analysis

We presented the frequency counts based on gender, age, and conditions validated. We also highlighted the relevant PROMs that are validated in MOH VDC conditions.

The psychometric properties including relevant cultural adaptations of each locally validated PROM will be included based on the COSMIN guidelines.

## Results

**Fig. 1:**
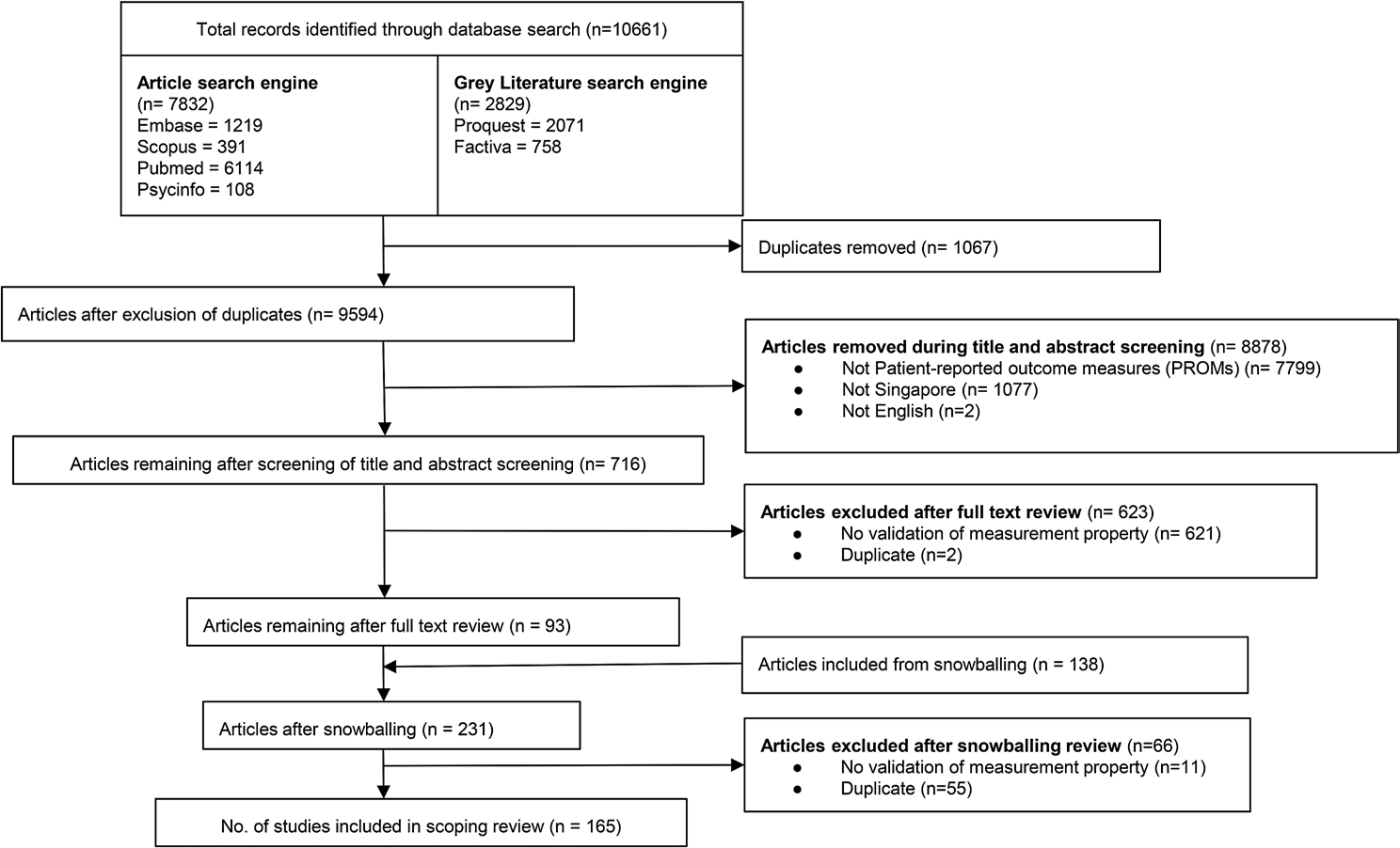
Scoping review flow chart

In total, 10,661 articles were retrieved from 6 databases. After removing 1067 duplicates and 8878 articles during title and abstract screening, 716 articles remained for full-text review. A total of 623 articles were further eliminated during full-text article screening, with reasons provided in Fig. 1. An additional hand-searching from the reference lists among the included articles produced another 138 articles, of which 72 relevant articles were accepted, giving us a final list of 165 articles for this study. The characteristics of the relevant studies are presented in Tables 1, 2 and 3.

**Table 1:** Study characteristics of included articles (*n* = 165) in Singapore

Study characteristics	Values, *n*
**Gender**	
F + M	158
F	7
**Mean age (years)**	
< 20	10
20–39	27
40–59	91
60–79	37
> 80	0
**Country**	
SG	158
SG + Multinational	7

**Table 2:** Type of disease-specific proms validated (*n* = 165) in Singapore

Type of disease-specific PROMs validated	Number of studies
EQ-5D-5L/EQ-5D/EQ-5D-Y DS	16
SF-36/SF-36v2/FACT-Cog/FACT-B/FACT-G/FACT-Ga/FACT/GOG-Ntx/FACT-N	9
PMHI/R-PMHI/SMPMHI	6
SCQOLS-10/SCQOLS-15/SCQOLS-D/WOMAC/Modified ShortMAC-F/KDQOL-SF/KDQOL-36/KDQOL-CF	5
HAQ/HAQ-DI/S-HAQ/PHQ-9/PHQ-1/PHQ-2/PHQ-A/WHOQOL-BREF/WHOQOL-AGE	4
ADDQoL/EORTC QLQ-C30/Quick-FLIC	3
VAS/OKS/VF-11/VF-14/KINDL-Kid (Singapore) and KINDL-Kiddo (Singapore)/RAI/CES-D/IPOS/PDQ-8/PAID/Sg-Paid-C/PDQ-39/HTN-SCP/CLDQ/CLDQ-SG/SLEQOL/SLEQOL-C/item bank to measure HRQoL	2
HUI3/Self-report measure of the extent of and reasons for medication nonadherence/DSC/LEFS/RAID/Constipation PROM that measures both symptom severity and constipation-related QoL/CAQ-B/PCS/BAI/MFSI-SF/MMAS/ThyPRO questionnaire/OIDP/DT/NRS/VRS/FPS-R/DRKA/C-MFPDI/RECAP/SAQOL-39 g/SAQOL-CSg/AOFAS/BASDAI/ASAS HI/EFAS/GIS/MDADI/CD-RISC10/PAQLQ/SMWEB/FMM and MFM/HQLQ/ASBQ/QIDS-SR16/ASQoL/SF-6D/WPAI/DHP-18/10-Item FAMCARE scale/DRNK/SF-12v2/ET/HADS/IVI/Screening questionnaires for identification of symptomatic KOA/Questionnaire for the Assessment of Pruritus/KOOS/Lequesne Algofunctional Index of knee/AF knowledge, attitude and perceptions questionnaire/PsAQoL/Lawton and Brody’s IADL/HFS-30/HFS-II/ACE Measure/CASP 11 SG scale/KCCQ/MPQ-SF/GPAQ/ASDAS-CRP/OSIS/OHS/ACDS/AADS/GDS-15/PRO-CTCAE/ReQoL-10 scale/SSc-QoL/MHC-SF/FoP-Q-SF/SQLS/SWEMWBS/MusiQoL/STED-QoL/QLQ-BR23/GIT 2.0/ACHC (Stoma) scale	1

Abbreviations: Male (M), female (F), Patient-reported outcome measures (PROMs), five-level EuroQoL Group’s five-dimension questionnaire (EQ-5D-5 L/EQ-5D), five-level EuroQoL Group’s five-dimension questionnaire descriptive system (EQ-5D-Y DS), 36-Item Short Form Health Survey / 36-Item Short Form Health Survey version 2 (SF-36/SF-36v2), Functional Assessment of Cancer Therapy: Cognitive Function (FACT-Cog), Functional Assessment of Cancer Therapy-Breast (FACT-B), Kansas City Cardiomyopathy Questionnaire (KCCQ), Functional Assessment of Cancer Therapy - General (FACT-G), Functional Assessment of Cancer Therapy-Gastric Module (FACT-Ga), Singapore Caregiver Quality of Life Scale − 10-item / 15-item (SCQOLS-10/SCQOLS-15), Singapore Caregiver Quality of Life Scale - Dementia (SCQOLS-D), Western Ontario and McMaster Universities Osteoarthritis Index (WOMAC), shortened version of the Western Ontario and McMaster Universities Osteoarthritis Index function scale (Modified ShortMAC-F), Kidney Disease Quality of Life Short Form (KDQOL-SF/KDQOL-36), Kidney Disease Quality of Life Cognitive Function subscale (KDQOL-CF), Health Assessment Questionnaire (HAQ), Scleroderma Health Assessment Questionnaire (S-HAQ), Health Assessment Questionnaire-Disability Index (HAQ-DI), nine-item Patient Health Questionnaire (PHQ-9), one-item Patient Health Questionnaire (PHQ-1), two-item Patient Health Questionnaire (PHQ-2), Rheumatology Attitudes Index (RAI), Rapid Positive Mental Health Instrument (R-PMHI), positive mental health instrument (PMHI), Center for Epidemiologic Studies Depression Scale (CES-D), Integrated Palliative care Outcome Scale (IPOS), Audit of Diabetes-Dependent Quality-of-Life (ADDQoL), World Health Organization Quality of Life-BREF (WHOQOL-BREF), European Organization for Research and Treatment of Cancer Quality of Life Questionnaire (EORTC QLQ-C30), Quality of Life Questionnaire – Breast Cancer Specific Module (QLQ-BR23), 8-item Parkinson’s Disease Questionnaire (PDQ-8), Problem Areas in Diabetes (PAID), Chinese version of Problem Areas In Diabetes Scale (Sg-Paid-C), 39-item Parkinson’s disease questionnaire (PDQ-39), Hypertension Self-Care Profile (HTN-SCP), Quick Version of the Functional Living Index-Cancer (Quick-FLIC), Health Utilities Index Mark 3 (HUI3), Dermatology Social Comparison (DSC), Lower Extremity Functional Scale (LEFS), Rheumatoid Arthritis Impact of Disease (RAID), Quality of Life (QoL), Childhood Asthma Questionnaire (CAQ-B), Pain Catastrophizing Scale (PCS), Health-Related Quality of Life (HRQoL), Beck Anxiety Inventory (BAI), Multidimensional Fatigue Syndrome Inventory- Short Form (MFSI-SF), Morisky Medication Adherence Scale (MMAS), Thyroid-specific quality of life questionnaire (ThyPRO questionnaire), Oral Impacts on Daily Performances (OIDP), Distress Thermometer (DT), Numerical Rating Scale (NRS), Visual Analogue Scale (VAS), Verbal Rating Scale (VRS), Faces Pain Scale-Revised (FPS-R), Chronic Liver Disease Questionnaire (CLDQ), Chronic Liver Disease Questionnaire - Singapore-Mandarin version (CLDQ-SG), Diabetic Retinopathy Knowledge and Attitudes (DRKA), Chinese Manchester foot pain and disability index (C-MFPDI), Recap of Atopic Eczema Patient-Reported Outcomes (RECAP), Stroke and Aphasia Quality of Life Scale (SAQOL-39 g) and its Mandarin adaptation (SAQOL-CSg), American Orthopaedic Foot and Ankle Society (AOFAS), Bath Ankylosing Spondylitis Disease Activity Index (BASDAI), Assessment of Spondyloarthritis International Society Health Index (ASAS HI), European Foot and Ankle Society (EFAS), Gout Impact Scale (GIS), MD Anderson Dysphagia Inventory (MDADI), Connor-Davidson Resilience Scale (CD-RISC10), Paediatric Asthma Quality of Life Questionnaire (PAQLQ), Singapore Mental Wellbeing (SMWEB), Medical Outcomes Study Family and Marital Functioning Measures (FMM and MFM), Hepatitis Quality of Life Questionnaire (HQLQ), Visual Function Index-11 (VF-11), Adult Sedentary Behaviour Questionnaire (ASBQ), 16-item Quick Inventory of Depressive Symptomatology – Self-Report (QIDS-SR16), Ankylosing Spondylitis quality of life (ASQoL), Systemic Lupus Erythematosus-Specific Quality-Of-Life instrument (SLEQOL), Systemic Lupus Erythematosus Quality of Life Questionnaire - Chinese version (SLEQOL-C), Short Form 6-Dimension (SF-6D), Short multidimensional positive mental health instrument (SMPMHI), Work Productivity and Activity Impairment (WPAI), Diabetes Health Profile-18 (DHP-18), Family Satisfaction with End-of-Life Care (10-Item FAMCARE scale), Diabetes-Related Nutrition Knowledge (DRNK), Short Form-12 version 2 (SF-12v2), Emotion Thermometer (ET), Hospital Anxiety and Depression Scales (HADS), Impact of Vision Impairment (IVI), Knee Osteoarthritis (KOA), Knee injury and Osteoarthritis Outcome Score (KOOS), Psoriatic Arthritis Quality of Life (PsAQoL), Instrumental Activities of Daily Living (IADL), Hemifacial Spasm-30 (HFS-30), Hypoglycemia Fear Survey-II (HFS-II), 8-item Altarum Consumer Engagement Measure™ (ACE Measure), Control, Autonomy, Self-realization, Pleasure Quality of Life scale (CASP‑11‑SG scale), Singapore (SG), Diabetes mellitus (DM), Hypertension (HTN), Atrial fibrillation (AF), Total Knee Replacement /Total Knee Arthroplasty (TKR/TKA), Value Driven Care (VDC), Oxford Knee Score (OKS), Short-Form of the McGill Pain Questionnaire (MPQ-SF), Global Physical Activity Questionnaire (GPAQ), Ankylosing Spondylitis Disease Activity Score with C-reactive protein (ASDAS-CRP), Oxford Hip Score (OHS), Oxford Shoulder Instability Score (OSIS), Asian Children Depression Scale (ACDS), Asian Adolescent Depression Scale (AADS), Geriatric Depression Screening Scale (GDS-15), Visual Function Index-14 (VF-14), U.S. National Cancer Institute’s Patient-Reported Outcomes version of the Common Terminology Criteria for Adverse Events (PRO-CTCAE), Recovering Quality of Life 10-item (ReQoL-10) scale, Systemic Sclerosis Quality of Life scale (SSc-QoL), 13-items World Health Organization Quality of Life Assessment-Older Adults Module (WHOQOL-AGE), Mental Health Continuum-Short Form (MHC-SF), Fear of Progression Questionnaire – Short Form (FoP-Q-SF), Schizophrenia Quality of Life Scale (SQLS), Acceptance of Chronic Health Conditions (ACHC), Functional Assessment of Cancer Therapy-Neutropenia (FACT-N), Short Warwick Edinburgh Mental Well-Being Scale (SWEMWBS), Patient Health Questionnaire for Adolescents (PHQ-A), Gastrointestinal Tract Instrument (GIT), Multiple sclerosis international quality of life questionnaire (MusiQoL), Singapore Thyroid Eye Disease Quality of Life questionnaire (STED-QoL) Value Driven Care (VDC), Oxford Knee Score (OKS), Short-Form of the McGill Pain Questionnaire (MPQ-SF), Global Physical Activity Questionnaire (GPAQ), Ankylosing Spondylitis Disease Activity Score with C-reactive protein (ASDAS-CRP), Oxford Hip Score (OHS), Oxford Shoulder Instability Score (OSIS), Asian Children Depression Scale (ACDS), Asian Adolescent Depression Scale (AADS), Geriatric Depression Screening Scale (GDS-15), Visual Function Index-14 (VF-14), U.S. National Cancer Institute’s Patient-Reported Outcomes version of the Common Terminology Criteria for Adverse Events (PRO-CTCAE), Recovering Quality of Life 10-item (ReQoL-10) scale, Systemic Sclerosis Quality of Life scale (SSc-QoL), 13-items World Health Organization Quality of Life Assessment-Older Adults Module (WHOQOL-AGE), Mental Health Continuum-Short Form (MHC-SF), Fear of Progression Questionnaire – Short Form (FoP-Q-SF), Schizophrenia Quality of Life Scale (SQLS), Acceptance of Chronic Health Conditions (ACHC), Functional Assessment of Cancer Therapy-Neutropenia (FACT-N), Short Warwick Edinburgh Mental Well-Being Scale (SWEMWBS), Patient Health Questionnaire for Adolescents (PHQ-A), Gastrointestinal Tract Instrument (GIT), Multiple sclerosis international quality of life questionnaire (MusiQoL), Singapore Thyroid Eye Disease Quality of Life questionnaire (STED-QoL)

**Table 3:** Type of diseases for which proms are validated (*n* = 165) in Singapore

Disease conditions including 19 VDC(*)	
General	45
Musculoskeletal diseases	13
Mental Health	8
DM*/Paediatrics	6
TKR/TKA*	5
Dermatological diseases/Gastrointestinal diseases	4
Caregiver-related/Visual impairment	3
Breast cancer*/Liver diseases/Parkinson’s disease/Thyroid diseases	2
AF/Chronic constipation/Colorectal resection*/Congestive heart failure*/End of life*/HTN/Ischemic stroke*/Kidney Disease/Neurological diseases/Total Hip Replacement / Total Hip Arthroplasty*	1
Acute myocardial infarction*/Caesarean section*/Cataract surgery*/Coronary artery bypass graft*/Hemorrhoidectomy*/Hernia repair*/Hysterectomy*/Laparoscopic cholecystectomy*/Pneumonia*/Spinal fusion*/Tonsillectomy*	0

The total number of PROMs identified was 115, with 70 being disease-specific PROMs and 45 being generic PROMs. While most were administered in English, selected PROMs were also translated and administered in other languages to cater to the local population. These include the World Health Organization Quality of Life (WHOQOL-BREF) questionnaire which is available in English, Chinese and Malay, and the EuroQol 5 Dimension questionnaire (EQ-5D) which is available in English, Chinese, Malay, and Tamil.

In Singapore, the most assessed PROMs based on the COSMIN psychometric properties include the EuroQol 5 Dimension questionnaire (EQ-5D) and the 36-Item Short Form Health Survey (SF-36), which were studied in 16 and 9 studies respectively. Of all the disease conditions, musculoskeletal diseases were supported by the largest number of PROMs (*n* = 13) found across multiple studies. We have also identified 16 additional diseases with validated PROMs beyond the 19 VDC conditions presently stipulated by MOH.

The common disease-specific PROMs identified from this study include the Western Ontario and McMaster Universities Osteoarthritis Index (WOMAC) / shortened version of the Western Ontario and McMaster Universities Osteoarthritis Index function scale (Modified ShortMAC-F) and Kidney Disease Quality of Life Short Form (KDQOL-SF/KDQOL-36/KDQOL-CF), each examined in 5 studies.

However, only 8 of the 19 MOH VDC conditions included PROMs that were found to be validated locally, and in languages such as English and Chinese. We also discovered that diseases such as hernia repair, caesarean section, and hemorrhoidectomy currently lack validated PROMs, limiting the ability to capture essential patient-reported outcomes.

The characteristics of all the validated disease-specific PROMs and their outcomes on the assessment of methodological quality and study quality in this study are summarised in Supplementary Table 2.

Of all the PROMs identified, internal consistency and construct validity were the most frequently assessed psychometric properties among all the conditions including the VDC conditions (summarised in Table 4). The consolidated repository of PROMs list from this study is collated in Supplementary Table 3.

**Table 4:** Number of studies (*n*= 165) that assessed the psychometric properties of each validated PROM in Singapore

(Disease conditions including 19 VDC (*)/ PROMS involved)/Psychometric properties	measurement error	internal consistency	test-retest reliability	inter-rater reliability	intra-rater reliability	Content validity	Criterion validity	Construct validity	Responsiveness	Interpretability (MCID)	Indication
Total Knee Replacement / Total Knee Arthroplasty*											
Modified ShortMAC-F		1						1		1	TKA
WOMAC		4	3					3	1	1	TKA, THA, OA
Lequesne Algofunctional Index of knee		1	1					1			TKR
KOOS		1	1					1			TKR
OKS		1						1		1	TKR
Total Hip Replacement / Total Hip Arthroplasty*											
OHS		1	1							1	THA
Congestive heart failure*											
KCCQ		1						1			Heart Failure
Ischemic stroke*											
SAQOL-39 g/ SAQOL-CSg		1	1					1			Stroke
Colorectal resection*											
ACHC (Stoma) scale		1	1			1		1			Colorectal Cancer
Breast cancer*											
FACT-B		1	2					1	2		Breast Cancer
QLQ-BR23		1					1				Breast Cancer
End of life*											
IPOS		2	2	2				2			Palliative Care, Heart Failure
Diabetes mellitus*											
PAID/Sg-Paid-C		2					1	2			DM
DHP-18		1						1			DM
DRKA			1				1	1			DM
DRNK		1	1			1		1			DM
ADDQoL		3	2			1		3	1		DM
HFS-II		1	1			1	1	1			DM
**Caregiver-related**											
SCQOLS-10/SCQOLS-15		3	3			1	1	2			Cancer, Heart Diseases
SCQOLS-D		2	2			2	1	1			Dementia
10-Item FAMCARE scale		1	1					1			Cancer
**Chronic constipation**											
Constipation PROM that measures both symptom severity and constipation-related QoL						1					Chronic Constipation
**General**											
EQ-5D-5 L/EQ-5D/EQ-5D-Y DS		1	7			2		12	4	2	Axial Spondyloarthritis, Rheumatic Disease, Cancer, Breast Cancer, ARMD, Parkinson’s Disease, Cataract Surgery, DM
SF-36/SF-36v2		7	5					6	1	1	Rheumatoid Arthritis, SLE, Axial Spondyloarthritis, Psoriatic Arthritis, THA
HUI3			1					1			Rheumatic Disease
Self-report measure of the extent of and reasons for medication nonadherence	1	1	1			1		1		1	DM
PCS		1						1			TKR
WHOQOL-BREF/WHOQOL-AGE		3	1				1	4			
item bank to measure HRQoL		1				2	1	1			
BAI		1					1	1			Breast Cancer
HADS		1					1				Cancer
DT							1	1			Cancer
LEFS	1	1				1	1	1			
MMAS		1					1	1			
HAQ/HAQ-DI		2	2				1	1			Psoriatic Arthritis, Axial Spondyloarthritis, Rheumatoid Arthritis
OIDP		1	1								
NRS		1	1				1				
VAS		1	1				1			1	Arthroscopic RCR
VRS		1	1				1				
FPS-R		1	1				1				
Quick-FLIC		3	3				2	3	2		Cancer
MDADI		1	1			1	1	1			Head and Neck Cancer
CD-RISC10	1	1	1			1		1		1	Axial Spondyloarthritis
FMM and MFM		1						1			SLE
PHQ-9/PHQ-1/PHQ-2		3					2	2			Depression, ASD
ASBQ			1					1			
QIDS-SR16		1					1	1			Depression
MFSI-SF		1						1	1		Breast Cancer, Lymphoma
SF-6D								1			
WPAI	1		1			1		1		1	Axial Spondyloarthritis
EORTC QLQ-C30		2					1	2			Cancer, Breast Cancer
SF-12v2						1	1	1			
ET		1					1				Cancer
Questionnaire for the Assessment of Pruritus			1			1	1	1			Uremic Pruritus
Lawton and Brody’s IADL		1						1			
CES-D		2		1				1			DM, Depression
ACE Measure		1					1	1			
CASP-11-SG scale		1						1			
FACT-G		1	1					2			Cancer
FACT-Cog		2	2				1	1	1	1	Breast Cancer
FACT-N		1						1			Chemotherapy-induced Neutropenia
MPQ-SF			1			1	1	1			Uremic Pruritus
GPAQ			1					1			
GDS-15		1	1	1			1				Depression
PRO-CTCAE						1					Breast and Colorectal Cancer
FoP-Q-SF		1	1				1				Cancer
FACT/GOG-Ntx		1						1	1		Cancer
**Paediatrics**											
KINDL-Kid (Singapore) and KINDL-Kiddo (Singapore)		2						1			DM
CAQ-B		1				1					Asthma
PAQLQ	1	1						1	1		Asthma
ACDS		1				1	1	1			Depression
AADS		1		1		1		1			Depression
PHQ-A		1						1			Depressive Disorders
**Kidney Disease**											
KDQOL-SF/KDQOL-36/KDQOL-CF		5					3	5	2		ESRD/Haemodialysis
**Dermatological diseases**											
DSC						1		1			Dermatology
RECAP	1	1						1			Atopic Eczema
SLEQOL/SLEQOL-C		1	1			2			1		SLE
HFS-30		1	1			1	1	1	1		Hemifacial Spasm
**Thyroid diseases**											
ThyPRO questionnaire		1				1		1	1		Thyroid/Graves’ Disease
STED-QoL							1				Thyroid Eye Disease
**Liver diseases**											
CLDQ/CLDQ-SG		2	1					1			Chronic Liver Disease
HQLQ		1	1					1			Hepatitis B
**Parkinson’s disease**											
PDQ-8		2	1					2			Parkinson’s Disease
PDQ-39		2	2			1		2			Parkinson’s Disease
**Gastrointestinal diseases**											
FACT-Ga		1						1			Gastric Cancer
S-HAQ			1					1			Systemic Sclerosis
SSc-QoL			1					1			Systemic Sclerosis
GIT 2.0		1	1					1			Systemic Sclerosis
**Musculoskeletal diseases**											
EFAS		1						1		1	Hallux Valgus
ASAS HI	1	1	1			1		1		1	Axial Spondyloarthritis
RAID		1	1			1		1			Rheumatoid Arthritis
BASDAI		1						1			Axial Spondyloarthritis
PsAQoL		1	1			1		1			Psoriatic Arthritis
ASDAS-CRP		1						1			Axial Spondyloarthritis
GIS		1						1			Gout
ASQoL		1	1			1		1			Ankylosing Spondylitis
RAI		2	2					2			SLE
AOFAS		1						1	1		Hallux Valgus
C-MFPDI		1	1			1	1				Inflammatory Arthritis
Screening questionnaires for identification of symptomatic KOA						1	1	1			KOA
OSIS										1	Arthroscopic Bankart Repair
**Visual impairment**											
IVI	1	1					1				Visual Impairment
VF-11		1				1	1	1			Visual Impairment
VF-14								1	1		Cataract Surgery
**Hypertension**											
HTN-SCP		2	2								HTN
**AF**											
AF knowledge, attitude and perceptions questionnaire		1				1		1			AF
**Mental Health**											
SMWEB		1						1			Mental Health
SMPMHI		1	1				1				Mental Health
R-PMHI		1					1	1			Mental Health
PMHI		4					4	2		1	Mental Health, Schizophrenia, Depression or Anxiety Spectrum Disorders
ReQoL-10 scale		1						1			Psychosis
MHC-SF		1					1	1			Mental Health
SQLS		1	1					1	1		Schizophrenia
SWEMWBS		1						1			Schizophrenia, Depression and Anxiety Spectrum Disorders
**Neurological diseases**											
MusiQoL		1				1		1			Multiple Sclerosis

Abbreviations: shortened version of the Western Ontario and McMaster Universities Osteoarthritis Index function scale (Modified ShortMAC-F), Western Ontario and McMaster Universities Osteoarthritis Index (WOMAC), Knee injury and Osteoarthritis Outcome Score (KOOS), Stroke and Aphasia Quality of Life Scale (SAQOL-39 g) and its Mandarin adaptation (SAQOL-CSg), Functional Assessment of Cancer Therapy-Breast (FACT-B), Integrated Palliative care Outcome Scale (IPOS), Problem Areas in Diabetes (PAID), Chinese version of Problem Areas In Diabetes Scale (Sg-Paid-C), Diabetes Health Profile-18 (DHP-18), Diabetic Retinopathy Knowledge and Attitudes (DRKA), Diabetes-Related Nutrition Knowledge (DRNK), Audit of Diabetes-Dependent Quality-of-Life (ADDQoL), Hypoglycemia Fear Survey-II (HFS-II), Singapore Caregiver Quality of Life Scale − 10-item / 15-item (SCQOLS-10/SCQOLS-15), Singapore Caregiver Quality of Life Scale - Dementia (SCQOLS-D), Family Satisfaction with End-of-Life Care (10-Item FAMCARE scale), Quality of Life (QoL), five-level EuroQoL Group’s five-dimension questionnaire (EQ-5D-5 L/EQ-5D), five-level EuroQoL Group’s five-dimension questionnaire descriptive system (EQ-5D-Y DS), 36-Item Short Form Health Survey / 36-Item Short Form Health Survey version 2 (SF-36/SF-36v2), Health Utilities Index Mark 3 (HUI3), Pain Catastrophizing Scale (PCS), World Health Organization Quality of Life-BREF (WHOQOL-BREF), Health-Related Quality of Life (HRQoL), Beck Anxiety Inventory (BAI), Hospital Anxiety and Depression Scales (HADS), Distress Thermometer (DT), Lower Extremity Functional Scale (LEFS), Morisky Medication Adherence Scale (MMAS), Health Assessment Questionnaire (HAQ), Scleroderma Health Assessment Questionnaire (S-HAQ), Health Assessment Questionnaire-Disability Index (HAQ-DI), Oral Impacts on Daily Performances (OIDP), Numerical Rating Scale (NRS), Visual Analogue Scale (VAS), Verbal Rating Scale (VRS), Faces Pain Scale-Revised (FPS-R), Quick Version of the Functional Living Index-Cancer (Quick-FLIC), MD Anderson Dysphagia Inventory (MDADI), Connor-Davidson Resilience Scale (CD-RISC10), Singapore Mental Wellbeing (SMWEB), Medical Outcomes Study Family and Marital Functioning Measures (FMM and MFM), nine-item Patient Health Questionnaire (PHQ-9), one-item Patient Health Questionnaire (PHQ-1), two-item Patient Health Questionnaire (PHQ-2), Adult Sedentary Behaviour Questionnaire (ASBQ), 16-item Quick Inventory of Depressive Symptomatology – Self-Report (QIDS-SR16), Multidimensional Fatigue Syndrome Inventory- Short Form (MFSI-SF), Short Form 6-Dimension (SF-6D), Work Productivity and Activity Impairment (WPAI), European Organization for Research and Treatment of Cancer Quality of Life Questionnaire (EORTC QLQ-C30), Quality of Life Questionnaire – Breast Cancer Specific Module (QLQ-BR23), Short Form-12 version 2 (SF-12v2), Emotion Thermometer (ET), Instrumental Activities of Daily Living (IADL), Center for Epidemiologic Studies Depression Scale (CES-D), 8-item Altarum Consumer Engagement Measure™ (ACE Measure), Rapid Positive Mental Health Instrument (R-PMHI), Control, Autonomy, Self-realization, Pleasure Quality of Life scale (CASP‑11‑SG scale), Functional Assessment of Cancer Therapy - General (FACT-G), Functional Assessment of Cancer Therapy: Cognitive Function (FACT-Cog), Childhood Asthma Questionnaire (CAQ-B), Paediatric Asthma Quality of Life Questionnaire (PAQLQ), Kidney Disease Quality of Life Short Form (KDQOL-SF/KDQOL-36), Kidney Disease Quality of Life Cognitive Function subscale (KDQOL-CF), Dermatology Social Comparison (DSC), Recap of Atopic Eczema Patient-Reported Outcomes (RECAP), Systemic Lupus Erythematosus-Specific Quality-Of-Life instrument (SLEQOL), Systemic Lupus Erythematosus Quality of Life Questionnaire - Chinese version (SLEQOL-C), Hemifacial Spasm-30 (HFS-30), Thyroid-specific quality of life questionnaire (ThyPRO questionnaire), Chronic Liver Disease Questionnaire (CLDQ), Chronic Liver Disease Questionnaire - Singapore-Mandarin version (CLDQ-SG), Hepatitis Quality of Life Questionnaire (HQLQ), 8-item Parkinson’s Disease Questionnaire (PDQ-8), 39-item Parkinson’s Disease Questionnaire (PDQ-39), Functional Assessment of Cancer Therapy-Gastric Module (FACT-Ga), European Foot and Ankle Society (EFAS), Assessment of Spondyloarthritis International Society Health Index (ASAS HI), Rheumatoid Arthritis Impact of Disease (RAID), Bath Ankylosing Spondylitis Disease Activity Index (BASDAI), Psoriatic Arthritis Quality of Life (PsAQoL), Gout Impact Scale (GIS), Ankylosing Spondylitis quality of life (ASQoL), Rheumatology Attitudes Index (RAI), American Orthopaedic Foot and Ankle Society (AOFAS), Chinese Manchester foot pain and disability index (C-MFPDI), Knee Osteoarthritis (KOA), Osteoarthritis (OA), Impact of Vision Impairment (IVI), Visual Function Index-11 (VF-11), Hypertension Self-Care Profile (HTN-SCP), Atrial fibrillation (AF), Singapore (SG), Diabetes mellitus (DM), Hypertension (HTN), Total Knee Replacement /Total Knee Arthroplasty (TKR/TKA), Value Driven Care (VDC), Systemic lupus erythematosus (SLE), Age-Related Macular Degeneration (ARMD), short multidimensional positive mental health instrument (SMPMHI), End stage renal disease (ESRD), Oxford Knee Score (OKS), Short-Form of the McGill Pain Questionnaire (MPQ-SF), positive mental health instrument (PMHI), Global Physical Activity Questionnaire (GPAQ), Ankylosing Spondylitis Disease Activity Score with C-reactive protein (ASDAS-CRP), Oxford Hip Score (OHS), Oxford Shoulder Instability Score (OSIS), Asian Children Depression Scale (ACDS), Asian Adolescent Depression Scale (AADS), Geriatric Depression Screening Scale (GDS-15), Minimal clinically important difference (MCID), Visual Function Index-14 (VF-14), Kansas City Cardiomyopathy Questionnaire (KCCQ), Total Hip Arthroplasty (THA), Arthroscopic Rotator Cuff Repair (Arthroscopic RCR), Functional Assessment of Cancer Therapy/Gynecologic Oncology Group—Neurotoxicity (FACT/GOG-Ntx), U.S. National Cancer Institute’s Patient-Reported Outcomes version of the Common Terminology Criteria for Adverse Events (PRO-CTCAE), Recovering Quality of Life 10-item (ReQoL-10) scale, Systemic Sclerosis Quality of Life scale (SSc-QoL), 13-items World Health Organization Quality of Life Assessment-Older Adults Module (WHOQOL-AGE), Mental Health Continuum-Short Form (MHC-SF), Fear of Progression Questionnaire – Short Form (FoP-Q-SF), Schizophrenia Quality of Life Scale (SQLS), Acceptance of Chronic Health Conditions (ACHC), Functional Assessment of Cancer Therapy-Neutropenia (FACT-N), Short Warwick Edinburgh Mental Well-Being Scale (SWEMWBS), Patient Health Questionnaire for Adolescents (PHQ-A), Gastrointestinal Tract Instrument (GIT), Autism Spectrum Disorder (ASD), Multiple sclerosis international quality of life questionnaire (MusiQoL), Singapore Thyroid Eye Disease Quality of Life questionnaire (STED-QoL)

## Discussion

To the best of our knowledge, this is the first study to compile a comprehensive list of validated PROMs in Singapore. It provides a foundational framework for identifying and critically reviewing locally validated PROMs in alignment with COSMIN guidelines [19] thereby supporting the nation’s efforts to advance patient-centred healthcare initiatives [20]. Among the generic PROMs identified, the EQ-5D stands out as the most widely validated locally. We hypothesise that its widespread use is due to its brevity and convenience, broad applicability across various morbidities, and the extensive research supporting it, which consistently yields comparable outcomes across studies [21, 22]. As such, we find that it is the standout generic PROM suitable for Singapore’s healthcare landscape as it is available in multiple languages and has demonstrated equivalence studies [23, 24]. 

Disease-specific PROMs such as Western Ontario and McMaster Universities Osteoarthritis Index (WOMAC) / shortened version of the Western Ontario and McMaster Universities Osteoarthritis Index function scale (Modified ShortMAC-F) and Kidney Disease Quality of Life Short Form (KDQOL-SF/KDQOL-36) appear to be the most ideal for use in the local context, with multiple studies supporting its use in Total Knee Replacement / Total Knee Arthroplasty and kidney disease respectively [25, 26]. 

Additionally, our findings indicate that musculoskeletal (MSK) diseases are the most prevalent condition with validated PROMs in Singapore. We postulate that this is due to the significant impact MSK pain has on physical aspects of quality of life (QOL), which tends to be more pronounced than in other disease categories [27, 28]. As such, the primary treatment goals for MSK conditions focus on pain reduction, functional improvement, and enhancing QOL [29–31]. 

In contrast, the emphasis is more on improving prognosis and reducing mortality risk in managing other conditions, such as ischemic stroke and breast cancer [32–35]. This distinction likely explains the increased emphasis on PROMs research within the MSK field, leading to a greater availability of validated PROMs for these conditions.

Despite the growing recognition of PROMs in evaluating healthcare interventions, there is a notable gap in validated PROMs studies for several VDC conditions. Currently, there are no validated PROM studies specific to the Singapore healthcare context for conditions such as hernia repair, caesarean section, and hemorrhoidectomy. The absence of validated PROMs for these conditions presents a challenge in accurately capturing patient-centred outcomes, limiting the ability to assess treatment effectiveness beyond traditional clinical metrics.

However, while some internationally available PROMs have been developed and validated according to the PROQOLID [14], such PROMs are yet to be validated locally. For instance, in hernia repair, PROMs such as the Hernia-related Quality-of-Life Survey (HerQLes) can assess the impact of hernias and their repair on patients’ quality of life [36]. In obstetric care, caesarean section outcomes are measured using PROMs such as the Obstetric Quality of Recovery-11 (ObsQoR-11) [37]. The impact of hemorrhoidectomy on daily life is evaluated through the Patient-reported outcome measure-haemorrhoidal impact and satisfaction score (PROM‐HISS) [38]. While these PROMs are widely used internationally, their validity and reliability in local healthcare settings remain uncertain.

In addition, our research uncovered the notable lack of studies focusing specifically on the 19 MOH VDC conditions. Furthermore, only 2 out of the 4 COSMIN psychometric properties were assessed considerably in the studies related to these validated PROMs. Altogether, these absences restrict healthcare providers from obtaining a holistic perspective of the impact of these conditions on patients, thereby potentially hindering patient-centric decision-making and appropriate care planning [39]. To address these gaps, further research is necessary to develop and assess PROMs for these underrepresented conditions, as well as to expand on covering all COSMIN psychometric properties. We urge researchers, healthcare professionals, and policymakers to collaborate in undertaking these efforts, ensuring that patient perspectives are effectively incorporated into healthcare delivery. Additionally, we plan to conduct studies to understand the barriers and facilitators of PROMs implementation to understand the gaps in the current implementation process. Henceforth, we aim to recommend strategies to smoothen the gaps in the PROMs implementation process. Such efforts would provide a more comprehensive assessment of patient experiences and support the ongoing advancement of patient-centred healthcare initiatives in routine clinical practice [40]. 

Our study differs from other research by focusing on locally validated PROMs in Singapore, an area that has been underexplored in the literature. This paper concentrates on PROMs validated specifically within the local context. For instance, we have identified PROMs that are suitable for all 4 of Singapore’s main cultures — Western, Chinese, Malay, and Indian [41]. Altogether, these findings provide valuable insights tailored to Singapore’s local demographic and contribute to the expanding body of evidence supporting the MOH’s VDC model in healthcare delivery.

With these studies, including relevant multinational research, neighbouring Southeast Asian (SEA) countries that share cultural, linguistic, and healthcare similarities with Singapore can leverage these findings to guide their selection of appropriate PROMs for relevant VDC conditions that may also exist there [42]. Consequently, adapting or adopting these PROMs can aid SEA nations in enhancing patient-centred care and improving healthcare outcome tracking.

The strengths of our study lie in its rigorous and comprehensive approach to identifying relevant articles on validated PROMs. In addition to a systematic database search, we expanded our search strategy by hand-searching the reference lists of included articles, as well as the references within those articles. This methodology allowed us to uncover both local studies and relevant multinational research, reducing the likelihood of excluding important articles. By employing this thorough search strategy, we were able to ensure that our findings reflect the most relevant and diverse range of studies, contributing a unique and valuable resource to the field that currently lacks a local PROMs repository. It provides a model for other countries looking to further develop their VDC conditions, especially in the aspect of VDC outcome assessment. Through creating their own local validated PROMs repository, the relevant stakeholders are able to either directly select and utilise the suitable validated PROMs or identify the missing gap in validated PROMs and take the necessary steps to validate PROMs for usage in VBC outcome assessment.

Despite the valuable insights provided by this study, several limitations should be acknowledged. First, only English full-text articles were included. However, there were only two foreign-language articles excluded during the full-text review. This will likely not impact our results as Singapore’s working language is English [33]. Additionally, the search strategy may have unintentionally excluded local studies that did not explicitly mention ‘Singapore’ in their titles or abstracts. Nevertheless, the inclusion of grey literature likely helped offset the potential gap created by reliance on only four article search engines.

## Conclusion

In conclusion, this scoping review provides an overview of locally validated PROMs for the MOH VDC conditions and other prevalent conditions in Singapore, evaluating their quality based on COSMIN psychometric properties for potential use in healthcare practices. As the first systematic compilation of such PROMs, this study fills a critical gap in the literature and offers valuable insights for future research and healthcare development. It also underscores the need for further research, particularly for conditions without validated PROMs. This highlights the importance of refining and validating PROMs tailored to the relevant patient population and improve healthcare outcome for patients.

## Supplementary Information

Below is the link to the electronic supplementary material.

Supplementary Material 1

Supplementary Material 2

Supplementary Material 3

## Data Availability

All data generated or analysed during this study are included in this published article and its supplementary information files.
