# Supplementary material for: A repository of Singapore validated PROMS: a scoping review
Source: J Patient Rep Outcomes. 2026 Feb 23;10:48. doi: 10.1186/s41687-026-01005-4 (PMC13031579; doi:10.1186/s41687-026-01005-4)
Supplement: Supplementary file 1 — Supplementary Material 1 [file 41687_2026_1005_MOESM1_ESM.docx]

| **No.** | **Embase** | **Scopus** | **PsycInfo** | **Pubmed** |
| --- | --- | --- | --- | --- |
| 1. 1 | ‘singapore'/exp OR singapore | "Singapore" | singapore | singapore |
|  | 'patient-reported outcome measure' OR 'patient-reported outcome measures' OR 'patient reported outcome measure' OR 'patient reported outcome measures' OR 'prom' OR 'proms' OR 'patient-reported-outcome measures' OR 'patient reported*' OR 'patient reported outcomes' OR 'patient-reported outcome assessment' OR 'patient-reported data' OR 'self-reported outcomes' | “Patient-reported outcome measure” OR “Patient-reported outcome measures” OR “Patient reported outcome measure” OR “Patient reported outcome measures” OR “PROM” OR “PROMs” OR “Patient-reported-outcome measures” OR “patient reported*” OR “Patient reported outcomes” OR “Patient-Reported Outcome Assessment” OR “Patient-Reported Data” OR “Self-Reported Outcomes” | 'Patient-reported outcome measure' OR 'Patient-reported outcome measures' OR 'Patient reported outcome measure' OR 'Patient reported outcome measures' OR 'PROM' OR 'PROMs' OR 'Patient-reported-outcome measures' OR 'patient reported*' OR 'Patient reported outcomes' OR 'Patient-Reported Outcome Assessment' OR 'Patient-Reported Data' OR 'Self-Reported Outcomes' | (Patient-reported outcome measure) OR (Patient-reported outcome measures) OR (Patient reported outcome measure) OR (Patient reported outcome measures) OR (PROM) OR (PROMs) OR (Patient-reported-outcome measures) OR (patient reported*) OR (Patient reported outcomes) OR (Patient-Reported Outcome Assessment) OR (Patient-Reported Data) OR (Self-Reported Outcomes) |
| 1. .. | 'intermethod comparison'/exp OR 'data collection method'/exp OR 'validation study'/exp OR 'feasibility study'/exp OR 'pilot study'/exp OR 'psychometry'/exp OR 'reproducibility'/exp OR reproducib*:ab,ti OR "audit":ab,ti OR psychometr*:ab,ti OR clinimetr*:ab,ti OR clinometr*:ab,ti OR "observer variation"/exp OR "observer variation":ab,ti OR "discriminant analysis"/exp OR "validity"/exp OR reliab*:ab,ti OR valid*:ab,ti OR "coefficient":ab,ti OR "internal consistency":ab,ti OR (cronbach*:ab,ti AND ("alpha":ab,ti OR "alphas":ab,ti)) OR "item correlation":ab,ti OR "item correlations":ab,ti OR "item selection":ab,ti OR "item selections":ab,ti OR "item reduction":ab,ti OR "item reductions":ab,ti OR "agreement":ab,ti OR "precision":ab,ti OR "imprecision":ab,ti OR "precise values":ab,ti OR "test–retest":ab,ti OR ("test":ab,ti AND "retest":ab,ti) OR (reliab*:ab,ti AND ("test":ab,ti OR "retest":ab,ti)) OR "stability":ab,ti OR "interrater":ab,ti OR "inter-rater":ab,ti OR "intrarater":ab,ti OR "intra-rater":ab,ti OR "intertester":ab,ti OR "inter-tester":ab,ti OR "intratester":ab,ti OR "intra-tester":ab,ti OR "interobeserver":ab,ti OR "inter-observer":ab,ti OR "intraobserver":ab,ti OR "intra-observer":ab,ti OR "intertechnician":ab,ti OR "intertechnician":ab,ti OR "intratechnician":ab,ti OR "intra-technician":ab,ti OR "interexaminer":ab,ti OR "inter-examiner":ab,ti OR "intraexaminer":ab,ti OR "intraexaminer":ab,ti OR "interassay":ab,ti OR "inter-assay":ab,ti OR "intraassay":ab,ti OR "intra-assay":ab,ti OR "interindividual":ab,ti OR "inter-individual":ab,ti OR "intraindividual":ab,ti OR "intra-individual":ab,ti OR "interparticipant":ab,ti OR "inter-participant":ab,ti OR "intraparticipant":ab,ti OR "intra-participant":ab,ti OR "kappa":ab,ti OR "kappas":ab,ti OR "coefficient of variation":ab,ti OR repeatab*:ab,ti OR (replicab*:ab,ti OR "repeated":ab,ti AND ("measure":ab,ti OR "measures":ab,ti OR "findings":ab,ti OR "result":ab,ti OR "results":ab,ti OR "test":ab,ti OR "tests":ab,ti)) OR generaliza*:ab,ti OR generalisa*:ab,ti OR "concordance":ab,ti OR ("intraclass":ab,ti AND correlation*:ab,ti) OR "discriminative":ab,ti OR "known group":ab,ti OR "factor analysis":ab,ti OR "factor analyses":ab,ti OR "factor structure":ab,ti OR "factor structures":ab,ti OR "dimensionality":ab,ti OR subscale*:ab,ti OR "multitrait scaling analysis":ab,ti OR "multitrait scaling analyses":ab,ti OR "item discriminant":ab,ti OR "interscale correlation":ab,ti OR "interscale correlations":ab,ti OR ("error":ab,ti OR "errors":ab,ti AND (measure*:ab,ti OR correlat*:ab,ti OR evaluat*:ab,ti OR "accuracy":ab,ti OR "accurate":ab,ti OR "precision":ab,ti OR "mean":ab,ti)) OR "individual variability":ab,ti OR "interval variability":ab,ti OR "rate variability":ab,ti OR "variability analysis":ab,ti OR ("uncertainty":ab,ti AND ("measurement":ab,ti OR "measuring":ab,ti)) OR "standard error of measurement":ab,ti OR sensitiv*:ab,ti OR responsive*:ab,ti OR ("limit":ab,ti AND "detection":ab,ti) OR "minimal detectable concentration":ab,ti OR interpretab*:ab,ti OR (small*:ab,ti AND ("real":ab,ti OR "detectable":ab,ti) AND ("change":ab,ti OR "difference":ab,ti)) OR "meaningful change":ab,ti OR "minimal important change":ab,ti OR "minimal important difference":ab,ti OR "minimally important change":ab,ti OR "minimally important difference":ab,ti OR "minimal detectable change":ab,ti OR "minimal detectable difference":ab,ti OR "minimally detectable change":ab,ti OR "minimally detectable difference":ab,ti OR "minimal real change":ab,ti OR "minimal real difference":ab,ti OR "minimally real change":ab,ti OR "minimally real difference":ab,ti OR "ceiling effect":ab,ti OR "floor effect":ab,ti OR "item response model":ab,ti OR "irt":ab,ti OR "rasch":ab,ti OR "differential item functioning":ab,ti OR "dif":ab,ti OR "computer adaptive testing":ab,ti OR "item bank":ab,ti OR "cross-cultural equivalence":ab,ti | “Validation studies” OR "Psychometr*" OR "Measurement propert*" OR "Validity" or "structural validity" or "hypothesis testing" OR "reliability" or "content validity" OR "internal consistency" OR "criterion validity" OR "cross cultural validity" OR "reproducibility" OR "responsiveness" OR "interpretability" OR "factor analysis" OR "coefficient" OR "correlation" OR "cronbach alpha" OR "qualitative adaptation" | 'Validation studies' OR 'Psychometr*' OR 'Measurement propert*' OR 'Validity' or 'structural validity' or 'hypothesis testing' OR 'reliability' or 'content validity' OR 'internal consistency' OR 'criterion validity' OR 'cross cultural validity' OR 'reproducibility' OR 'responsiveness' OR 'interpretability' OR 'factor analysis' OR 'coefficient' OR 'correlation' OR 'cronbach alpha' OR 'qualitative adaptation' | instrumentation[sh] OR methods[sh] OR Validation Studies OR Comparative Study OR “psychometrics”[MeSH] OR psychometr*[tiab] OR clinimetr*[tw] OR clinometr*[tw] OR outcome assessment[tiab] OR outcome measure*[tw] OR “Health Status Indicators”[Mesh] OR “reproducibility of results”[MeSH] OR reproducib*[tiab] OR “discriminant analysis”[MeSH] OR reliab*[tiab] OR unreliab*[tiab] OR valid*[tiab] OR coefficient[tiab] OR homogeneity[tiab] OR homogeneous[tiab] OR “internal consistency”[tiab] OR (cronbach*[tiab] AND (alpha[tiab] OR alphas[tiab])) OR (item[tiab] AND (correlation*[tiab] OR selection*[tiab] OR reduction*[tiab])) OR agreement[tiab] OR precision[tiab] OR imprecision[tiab] OR “precise values”[tiab] OR test–retest[tiab] OR (test[tiab] AND retest[tiab]) OR (reliab*[tiab] AND (test[tiab] OR retest[tiab])) OR stability[tiab] OR interrater[tiab] OR inter-rater[tiab] OR intrarater[tiab] OR intra-rater[tiab] OR interobserver[tiab] OR inter-observer[tiab] OR intraobserver[tiab] OR intra-observer[tiab] OR interindividual[tiab] OR inter-individual[tiab] OR intraindividual[tiab] OR intra-individual[tiab] OR interparticipant[tiab] OR inter-participant[tiab] OR intraparticipant[tiab] OR intra-participant[tiab] OR kappa[tiab] OR kappa’s[tiab] OR kappas[tiab] OR repeatab*[tiab] OR ((replicab*[tiab] OR repeated[tiab]) AND (measure[tiab] OR measures[tiab] OR findings[tiab] OR result[tiab] OR results[tiab] OR test[tiab] OR tests[tiab])) OR generaliza*[tiab] OR generalisa*[tiab] OR (intraclass[tiab] AND correlation*[tiab]) OR discriminative[tiab] OR “known group”[tiab] OR factor analysis[tiab] OR factor analyses[tiab] OR dimension*[tiab] OR subscale*[tiab] OR (multitrait[tiab] AND scaling[tiab] AND (analysis[tiab] OR analyses[tiab])) OR item discriminant[tiab] OR interscale correlation*[tiab] OR error[tiab] OR errors[tiab] OR “individual variability”[tiab] OR (variability[tiab] AND (analysis[tiab] OR values[tiab])) OR (uncertainty[tiab] AND (measurement[tiab] OR measuring[tiab])) OR “standard error of measurement”[tiab] OR sensitiv*[tiab] OR responsive*[tiab] OR ((minimal[tiab] OR minimally[tiab] OR clinical[tiab] OR clinically[tiab]) AND (important[tiab] OR significant[tiab] OR detectable[tiab]) AND (change[tiab] OR difference[tiab])) OR (small*[tiab] AND (real[tiab] OR detectable[tiab]) AND (change[tiab] OR difference[tiab])) OR meaningful change[tiab] OR “ceiling effect”[tiab] OR “floor effect”[tiab] OR “Item response model”[tiab] OR IRT[tiab] OR Rasch[tiab] OR “Differential item functioning”[tiab] OR DIF[tiab] OR “computer adaptive testing”[tiab] OR “item bank”[tiab] OR “cross-cultural equivalence”[tiab] |
|  | 'quality of life' OR 'health-related quality of life' | “quality of life” OR “health-related quality of life” | 'Quality of life' OR 'Health-related quality of life' | (quality of life) OR (health-related quality of life) |
|  | #1 AND (#2 OR #4) AND #3 | #1 AND (#2 OR #4) AND #3 | #1 AND (#2 OR #4) AND #3 | #1 AND (#2 OR #4) AND #3 |

| **No.** | **Proquest** | **Factiva** |
| --- | --- | --- |
|  | Singapore | “Singapore” |
|  | "patient-reported outcome measure" OR "patient-reported outcome measures" OR "patient reported outcome measure" OR "patient reported outcome measures" OR "prom" OR "proms" OR "patient-reported-outcome measures" OR "patient reported" OR "patient reported outcomes" OR "patient-reported outcome assessment" OR "patient-reported data" OR "self-reported outcomes” | "patient-reported outcome measure" OR "patient-reported outcome measures" OR "patient reported outcome measure" OR "patient reported outcome measures" OR "prom" OR "proms" OR "patient-reported-outcome measures" OR "patient reported" OR "patient reported outcomes" OR "patient-reported outcome assessment" OR "patient-reported data" OR "self-reported outcomes" |
|  | “Validation studies” OR "Psychometric" OR "Psychometrics" OR "Measurement properties" OR "Measurement property" OR "Validity" or "structural validity" or "hypothesis testing" OR "reliability" or "content validity" OR "internal consistency" OR "criterion validity" OR "cross cultural validity" OR "reproducibility" OR "responsiveness" OR "interpretability" OR "factor analysis" OR "coefficient" OR "correlation" OR "cronbach alpha" OR "qualitative adaptation" | "Validation studies" OR "Psychometric" OR "Psychometrics" OR "Measurement properties" OR "Measurement property" OR "Validity" or "structural validity" or "hypothesis testing" OR "reliability" or "content validity" OR "internal consistency" OR "criterion validity" OR "cross cultural validity" OR "reproducibility" OR "responsiveness" OR "interpretability" OR "factor analysis" OR "coefficient" OR "correlation" OR "cronbach alpha" OR "qualitative adaptation" |
|  | “quality of life” OR “health-related quality of life” | “quality of life” OR “health-related quality of life” |
|  | #1 AND (#2 OR #4) AND #3 | #1 AND (#2 OR #4) AND #3 |
