# Supplementary material for: A repository of Singapore validated PROMS: a scoping review
Source: J Patient Rep Outcomes. 2026 Feb 23;10:48. doi: 10.1186/s41687-026-01005-4 (PMC13031579; doi:10.1186/s41687-026-01005-4)
Supplement: Supplementary file 2 — Supplementary Material 2 [file 41687_2026_1005_MOESM2_ESM.docx]

| **Title** | **Gender** | **Number of subjects** | **Mean age (years)** | **PROMs** | **Language (s)** | **Country** | **(Conditions) psychometric properties** | **Culturally adapted (qualitative)** | **Remark** |
| --- | --- | --- | --- | --- | --- | --- | --- | --- | --- |
| A Chinese version of the Rheumatology Attitudes Index is a valid and reliable measure of learned helplessness in patients with SLE | F + M | 69 | 32.1 | RAI | Chinese | SG | (SLE) construct validity, internal consistency, test-test reliability | X | Adequate |
| A community-based study of scaling assumptions and construct validity of the English (UK) and Chinese (HK) SF-36 in Singapore | F + M | 5503 | majority 31-40 | SF-36 | English, Chinese | SG | construct validity, internal consistency, test-retest reliability | ✔️ | Very good |
| A Comparison of the EuroQol-5D and the Health Utilities Index Mark 3 in Patients with Rheumatic Disease | F + M | 114 | 49 | EQ-5D and HUI3 | English, Chinese | SG | (Rheumatic Disease) Test-retest reliability, construct validity | ✔️ | Very good |
| A cultural adaptation and validation study of a self report measure of the extent of and reasons for medication nonadherence among patients with diabetes in Singapore | F + M | 393 | 59.4 | self-report measure of the extent of and reasons for medication nonadherence | English, Chinese, Malay | SG | (DM) Interpretability, content validity, internal consistency, test–retest reliability, construct validity, measurement error | ✔️ | Very good |
| A questionnaire for the assessment of pruritus: validation in uremic patients | F + M | 145 | 62 | Questionnaire for the Assessment of Pruritus, MPQ-SF | English | SG | (Uremic Pruritus) validity (content validity, criterion validity, and construct validity), test-retest reliability | ✔️ | Adequate |
| Adaptation of Chinese and English versions of the Ankylosing Spondylitis quality of life (ASQoL) scale for use in Singapore | F + M | 183 | 39.4 | ASQoL | English, Chinese | SG | (Ankylosing Spondylitis) internal consistency, test-retest reliability, construct validity, content validity | ✔️ | Very good |
| Adaptation of Chinese and English versions of the Psoriatic Arthritis Quality of Life (PsAQoL) scale for use in Singapore | F + M | 98 | 51.5 | PsAQoL | English, Chinese | SG | (Psoriatic Arthritis) internal consistency, Test–retest reliability, content validity, construct validity | ✔️ | Adequate |
| An evaluation of the reliability and validity of the visual functioning questionnaire (VF-11) using Rasch analysis in an Asian population | F + M | 618 | 65.4 | VF-11 | English, Malay | SG | (Visual Impairment) internal consistency, criterion validity, content validity, construct validity | ✔️ | Very good |
| Are English and Chinese Versions of the Audit of Diabetes-Dependent Quality of Life Equivalent? An Exploratory Study Based on the Universalist Approach | F + M | 68 | 54.3 | ADDQOL | English, Chinese | SG | (DM) construct validity^m^, internal consistency, test-retest reliability, responsiveness | X | Adequate |
| Are English- and Chinese-language versions of the SF-6D equivalent? A comparison from a population-based study | F + M | 2558 | 40.4 | SF-6D | English, Chinese | SG | construct validity^m^ | ✔️ | Very good |
| Are Oxford Hip Score and Western Ontario and McMaster Universities Osteoarthritis Index Useful Predictors of Clinical Meaningful Improvement and Satisfaction After Total Hip Arthroplasty? | F + M | 1334 | 61.4 | OHS, WOMAC, SF-36 | English | SG | (THA) internal consistency, test-retest reliability, interpretability | X |  |
| Asian adaptation and validation of an English version of the multiple sclerosis international quality of life questionnaire (MusiQoL) | F + M | 81 | 42.7 | MusiQoL | English | SG + M | (Multiple Sclerosis) internal consistency, content validity, construct validity | X | Very good |
| Assessing the Content Validity of the EQ-5D Questionnaire Among Asians in Singapore: A Qualitative Study | F + M | 60 | 58.9 | EQ-5D | English, Chinese | SG | content validity | ✔️ |  |
| Assessment of the psychometric properties of the Chinese Impact of Vision Impairment questionnaire in a population-based study: findings from the Singapore Chinese Eye Study | F + M | 3353 | 59.7 | IVI | English, Chinese | SG | (Visual Impairment) criterion validity, reliability (internal consistency and measurement error) | ✔️ |  |
| Audit of Diabetes-Dependent Quality of Life (ADDQoL) [Chinese version for Singapore] questionnaire | F + M | 88 | 56.6 | ADDQoL | Chinese | SG | (DM) internal consistency, test-retest reliability, construct validity | ✔️ | Adequate |
| Botulinum toxin improves quality of life in hemifacial spasm: validation of a questionnaire (HFS-30) | F + M | 80 | 56.3 | HFS-30 | English | SG | (Hemifacial Spasm) internal consistency, test-retest reliability, validity (content validity, criterion validity, and construct validity), responsiveness | X | Adequate |
| Can the Lower Extremity Functional Scale Be Used in Children and Adolescents? A Validation Study | F + M | 178 | 13 | LEFS | English | SG | internal consistency, measurement error, content validity, criterion validity, construct validity | X | Very good |
| Clinical validation of the chronic liver disease questionnaire for the Chinese population in Singapore | F + M | 242 | 67 | CLDQ-SG | Chinese | SG | (Chronic Liver Disease) internal consistency, construct validity | ✔️ | Very good |
| Comparison of the measurement properties between a short and generic instrument, the 5-level EuroQoL Group's 5-dimension (EQ-5D-5L) questionnaire, and a longer and disease-specific instrument, the Functional Assessment of Cancer Therapy-Breast (FACT-B), in Asian breast cancer patients | F + M | 269 | 52.1 | EQ-5D-5L, FACT-B | English, Chinese | SG | (Breast Cancer) test-retest reliability, responsiveness | X |  |
| Conceptualization And Manifestation Of Depression In An Asian Context: Formal Construction And Validation Of A Children’S Depression Scale In Singapore | F + M | 442 | majority 11-12 | ACDS | English | SG | (Depression) content validity, construct validity, criterion validity, internal consistency | ✔️ | Very good |
| Criterion-based validity and reliability of the Geriatric Depression Screening Scale (GDS-15) in a large validation sample of community-living Asian older adults | F + M | 4253 | 73.8 | GDS-15 | English, Chinese, Malay | SG | (Depression) criterion validity, test-retest reliability, inter-rater reliability, internal consistency | ✔️ |  |
| Cross cultural adaptation and validation of the Chinese Health Assessment Questionnaire for use in rheumatoid arthritis | F + M | 42 | majority 40-49 | HAQ | Chinese | SG | (Rheumatoid Arthritis) internal consistency, test-retest reliability, criterion validity | ✔️ |  |
| Cross-cultural adaptation and content validation of the Singapore English version of EQ-5D-Y: a qualitative study | F + M | 14 | 10.4 | EQ-5D-Y DS | English | SG | content validity | ✔️ |  |
| Cross-cultural adaptation and validation of Singapore English and Chinese versions of the Knee injury and Osteoarthritis Outcome Score (KOOS) in Asians with knee osteoarthritis in Singapore | F + M | 258 | 66 | KOOS | English, Chinese | SG | (TKR) internal consistency, test–retest reliability, construct validity | ✔️ | Very good |
| Cross-cultural adaptation and validation of Singapore English and Chinese versions of the Lequesne Algofunctional Index of knee in Asians with knee osteoarthritis in Singapore | F + M | 258 | 66 | Lequesne Algofunctional Index of knee | English, Chinese | SG | (TKR) internal consistency, test–retest reliability, construct validity | ✔️ | Very good |
| Cross-cultural adaptation and validation of Singapore English and Chinese Versions of the Oxford Knee Score (OKS) in knee osteoarthritis patients undergoing total knee replacement | F + M | 258 | 66 | OKS | English, Chinese | SG | (TKR) internal consistency, construct validity | ✔️ | Very good |
| Cross-cultural adaptation and validation of Singapore Malay and Tamil versions of the EQ-5D | F + M | 176 | 52.6 | EQ-5D | Malay, Tamil | SG | construct validity | ✔️ | Very good |
| Cross-cultural adaptation of the Systemic Lupus Erythematosus Quality of Life Questionnaire into Chinese | F + M | 237 | 47.63 | SLEQOL-C | Chinese | SG | (SLE) content validity | ✔️ |  |
| Cross-cultural measurement equivalence of the 5-level EQ-5D (EQ-5D-5L) in patients with type 2 diabetes mellitus in Singapore | F + M | 729 | 56.81 | EQ-5D-5L | English, Chinese, Malay | SG | (DM) construct validity^m^ | X | Adequate |
| Cross-cultural measurement equivalence of the EQ-5D-5L items for English-speaking Asians in Singapore | F + M | 257 | 39.1 | EQ-5D-5L | English | SG | Interpretability, construct validity^m^ | ✔️ | Very good |
| Cultural adaptation and validation of a questionnaire for use in hepatitis B patients | F + M | 298 | 44.14 | HQLQ | English | SG | (Hepatitis B) internal consistency, test-retest reliability, construct validity | ✔️ | Very good |
| Defining minimal clinically important difference, patient acceptable symptomatic state and substantial clinical benefit for the visual analog scale pain score after arthroscopic rotator cuff repair | F + M | 286 | 60.2 | VAS | English | SG | (Arthroscopic RCR) interpretability | X |  |
| Derivation, and establishment of the validity and reliability, of the CASP-11-SG quality of life scale among community-dwelling older adults | F + M | 3526 | 71 | CASP‑11‑SG scale | English, Chinese | SG | internal consistency, construct validity | ✔️ | Adequate |
| Determining the Minimal Clinically Important Difference on the Oxford Shoulder Instability Score in Patients Undergoing Arthroscopic Bankart Repair for Shoulder Instability | F + M | 79 | 29.9 | OSIS | English | SG | (Arthroscopic Bankart Repair) interpretability | X |  |
| Developing item banks to measure three important domains of health-related quality of life (HRQOL) in Singapore | F + M | 45 | 45.1 | item bank to measure HRQOL | English | SG | content validity | ✔️ |  |
| Development and calibration of a novel social relationship item bank to measure health-related quality of life (HRQoL) in Singapore | F + M | 503 | majority ≥50 | item bank to measure HRQoL | English, Chinese | SG | criterion validity, content validity, construct validity, internal consistency, | ✔️ | Very good |
| Development and evaluation of a quality of life measurement scale in English and Chinese for family caregivers of patients with advanced cancers | F + M | 612 | 48 | SCQOLS | English, Chinese | SG | (Advanced Cancers) internal consistency, test-retest reliability, construct validity | ✔️ | Doubtful |
| Development and evaluation of the Singapore Caregiver Quality of Life Scale - Dementia | F + M | 102 | 55 | SCQOLS-D | English | SG | (Dementia) construct validity, content validity, test-retest reliability, internal consistency | X | Very good |
| Development and preliminary validation of a systemic lupus erythematosus-specific quality-of-life instrument (SLEQOL) | F + M | 275 | 40.1 | SLEQOL | English | SG | (SLE) internal consistency, test-retest reliability, content validity, responsiveness | ✔️ |  |
| Development and validation of a depression scale for Asian adolescents | F + M | 480 | 15.8 | AADS | English | SG | (Depression) content validity, construct validity, internal consistency, inter-rater reliability | ✔️ | Very good |
| Development and Validation of a Mental Wellbeing Scale in Singapore | F + M | 1092 | 30.5 | SMWEB | English, Chinese, Malay, Tamil | SG | (Mental Health) internal consistency, construct validity | ✔️ | Very good |
| Development and validation of the Dermatology Social Comparison (DSC) Scale | F + M | 1053 | 43.5 | DSC | English | SG | (Dermatology) construct validity, content validity | ✔️ | Very good |
| Development and validation of the Rapid Positive Mental Health Instrument (R-PMHI) for measuring mental health outcomes in the population | F + M | 2975 | 42.1 | R-PMHI | English | SG | (Mental Health) construct validity, criterion validity, Internal consistency | ✔️ | Adequate |
| Development and Validation of the Singapore Thyroid Eye Disease Quality of Life Questionnaire | F + M | 20 | 49 | STED-QoL | English | SG | (Thyroid Eye Disease) criterion validity | ✔️ |  |
| Development of a diabetes-related nutrition knowledge questionnaire for individuals with type 2 diabetes mellitus in Singapore | F + M | 100 | 52 | DRNK | English | SG | (DM) internal consistency, content validity, construct validity, test-retest reliability | ✔️ | Very good |
| Development of a short form of the Singapore Caregiver Quality of Life Scale – Dementia: SCQOLS-D-15 | F + M | 102 | 54.6 | SCQOLS-D | English | SG | (Dementia) criterion validity, Internal consistency, test-retest reliability, content validity | ✔️ | Very good |
| Development of the Chinese, Malay and Tamil translations of the Positive Mental Health Instrument: Cross-cultural adaptation, validity and internal consistency | F + M | 48 | 50.8 | PMHI | Malay, Chinese, Tamil | SG | (Mental Health) criterion validity, internal consistency | ✔️ |  |
| Development of the Inpatient Dignity Scale Through Studies in Japan, Singapore, and the United Kingdom | F + M | 363 | 41.4 | IPDS | English | SG + M | internal consistency, criterion validity, construct validity, content validity | ✔️ | Very good |
| Development, validity and reliability of the short multidimensional positive mental health instrument | F + M | 201 | 41 | SMPMHI | English | SG | (Mental Health) internal consistency, criterion validity, test–retest reliability | X | Very good |
| Diabetes Health Profile-18 is Reliable, Valid and Sensitive in Singapore | F + M | 204 | 45.4 | DHP-18 | English | SG | (DM) internal consistency, construct validity | X | Very good |
| Do English and Chinese EQ-5D versions demonstrate measurement equivalence? an exploratory study | F + M | 114 | 49.5 | EQ-5D | English, Chinese | SG | construct validity^m^ | ✔️ | Very good |
| Domain-Specific Adult Sedentary Behaviour Questionnaire (ASBQ) and the GPAQ Single-Item Question: A Reliability and Validity Study in an Asian Population | F + M | 84 | 32.5 | GPAQ, ASBQ | English | SG | construct validity, test-retest reliability | ✔️ | Doubtful |
| Effect of Context Specificity on Response to the Shortened WOMAC Function Scale in Patients Undergoing Total Knee Arthroplasty | F | 114 | 66.4 | Modified ShortMAC-F | English | SG | (TKR / TKA) internal consistency, construct validity, interpretability | X | Very good |
| Evaluation of the properties of the rheumatoid arthritis impact of disease (RAID) score in multiethnic asian patients with rheumatoid arthritis | F + M | 82 | 53 | RAID | English | SG | (Rheumatoid Arthritis) content validity, construct validity, internal consistency, test-retest reliability | X | Adequate |
| Factor structure of the 10-item CES-D scale among community dwelling older adults in Singapore | F + M | 1013 | 71.5 | CES-D | English | SG | (Depression) internal consistency, construct validity | ✔️ | Adequate |
| Factor structure of the Singapore English version of the KINDL children quality of life questionnaire | F + M | 328 | 9.6 | KINDL-Kid (Singapore) and KINDL-Kiddo (Singapore) | English | SG | internal consistency | X |  |
| Involving patients in the process: Development of a constipation patient-reported outcome measure for symptoms and quality of life | F + M | 15 | 53 | constipation PROM that measures both symptom severity and constipation-related QoL | English | SG | (Chronic Constipation) content validity | ✔️ |  |
| Is EQ-5D a valid quality of life instrument in patients with Parkinsons disease? A study in Singapore | F + M | 208 | 62.1 | EQ-5D | English, Chinese | SG | (Parkinson's Disease) construct validity | X | Adequate |
| Is the childhood asthma questionnaire a good measure of health-related quality of life of asthmatic children in Asia?: validation among paediatric patients with asthma in Singapore | F + M | 96 | 8.7 | CAQ-B | English | SG | (Asthma) content validity, Internal consistency | ✔️ |  |
| Linguistic validation of the simplified Chinese version of the US National Cancer Institute’s patient-reported outcomes version of the common terminology criteria for adverse events (PRO-CTCAE™) | F + M | 96 | 55.16 | PRO-CTCAE | Chinese | SG | (Breast and Colorectal Cancer) content validity | ✔️ |  |
| Measurement equivalence of the English, Chinese and Malay versions of the World Health Organization quality of life (WHOQOL-BREF) questionnaires | F + M | 1203 | 50.5 | WHOQOL-BREF | English, Chinese, Malay | SG | construct validity^m^ | ✔️ | Very good |
| Measurement properties of pain catastrophizing scale in patients with knee osteoarthritis | F + M | 675 | 65.52 | PCS | English | SG | (TKR) internal consistency, construct validity | ✔️ | Very good |
| Measurement properties of the 15-Item Singapore Caregiver Quality of Life Scale (SCQOLS-15) in family caregivers of patients with heart diseases | F + M | 327 | 49 | SCQOLS-15 | English, Chinese | SG | (Heart Diseases) internal consistency, construct validity, test-retest reliability, criterion validity | X | Very good |
| Measurement Properties of the Chinese Language Version of the Functional Assessment of Cancer Therapy General in a Singaporean Population | F + M | 165 | 54 | FACT-G | Chinese | SG | (Cancer) construct validity, internal consistency, test-retest reliability | ✔️ | Very good |
| Measurement properties of the Chinese Version of the Kidney Disease Quality of Life-Short Form (KDQOL-SF™) in end-stage renal disease patients with poor prognosis in Singapore | F + M | 78 | 76 | KDQOL-SF | Chinese | SG | (ESRD) criterion validity, construct validity, responsiveness, Internal consistency | ✔️ | Adequate |
| Measurement properties of the Chinese version of the Kidney Disease Quality of Life-Short Form (KDQOL-SFTM) in end-stage renal disease patients with poor prognosis in singapore | F + M | 78 | 76 | KDQOL-SF | Chinese | SG | (ESRD) internal consistency, criterion validity, responsiveness, construct validity | ✔️ | Adequate |
| Measurement properties of the English and Chinese versions of the Functional Assessment of Cancer Therapy-Breast (FACT-B) in Asian breast cancer patients | F | 271 | 52.65 | FACT-B | English, Chinese | SG | (Breast Cancer) internal consistency, test-retest reliability, construct validity, responsiveness | ✔️ | Very good |
| Measuring quality of life in Chinese cancer patients: a new version of the Functional Living Index for Cancer (Chinese) | F + M | 152 | 53 | FLIC | Chinese | SG | (Cancer) construct validity, reliability (internal consistency, measurement error) | X | Very good |
| Minimal clinically important difference (MCID) for the functional assessment of cancer therapy: Cognitive function (FACT-Cog) in breast cancer patients | F | 220 | 50.9 | FACT-Cog | English, Chinese | SG | (Breast Cancer) test-retest reliability, internal consistency, responsiveness, interpretability | X |  |
| Patient-reported outcomes in heart failure with preserved vs. reduced ejection fraction: focus on physical independence | F + M | 5391 | 62.1 | KCCQ | English | SG + M | (Heart Failure) internal consistency, construct validity | X | Very good |
| Pediatric asthma quality of life questionnaire: validation in children from Singapore | F + M | 47 | 9.9 | PAQLQ | English | SG | (Asthma) reliability (internal consistency, measurement error), construct validity, responsiveness | ✔️ | Doubtful |
| Physical and Cognitive Domains of the Instrumental Activities of Daily Living: Validation in a Multiethnic Population of Asian Older Adults | F + M | 1072 | 69.3 | Lawton and Brody's IADL | English, Chinese, Malay | SG | internal consistency, construct validity | ✔️ | Very good |
| Preliminary Validation of the HS-QoL: A Quality-of-Life Measure for Hidradenitis Suppurativa | F + M | 55 | 34.5 | HS-QoL | English | SG + M | (HS) internal consistency, construct validity | ✔️ | Adequate |
| Psychometric evaluation of the 8-item Altarum Consumer Engagement (ACE) Measure™ in community-dwelling adults in Singapore | F + M | 400 | 49.7 | ACE Measure | English | SG | construct validity, criterion validity, internal consistency | ✔️ | Adequate |
| Psychometric evaluation of the Schizophrenia Quality of Life Scale (SQLS) in English- and Chinese-speaking Asians in Singapore | F + M | 202 | 37.8 | SQLS | English, Chinese | SG | (Schizophrenia) responsiveness, internal consistency, construct validity, test-retest reliability | X | Very good |
| Psychometric properties and measurement equivalence of the English and Chinese versions of the Beck Anxiety Inventory in patients with breast cancer | F | 244 | 50.83 | BAI | English, Chinese | SG | (Breast Cancer) internal consistency, criterion validity, construct validity^m^ | ✔️ | Very good |
| Psychometric Properties and Measurement Equivalence of the English and Chinese Versions of the Functional Assessment of Cancer Therapy-Cognitive in Asian Patients With Breast Cancer | F | 328 | 51.8 | FACT-Cog | English, Chinese | SG | (Breast Cancer) internal consistency, test-retest reliability, criterion validity, construct validity^m^ | ✔️ | Very good |
| Psychometric properties and measurement equivalence of the Multidimensional Fatigue Syndrome Inventory- Short Form (MFSI-SF) amongst breast cancer and lymphoma patients in Singapore | F + M | 246 | 53.4 | MFSI-SF | English, Chinese | SG | (Breast Cancer, Lymphoma) internal consistency, construct validity^m^, responsiveness | X | Adequate |
| Psychometric properties and population norms of the positive mental health instrument in a representative multi-ethnic Asian population | F + M | 1925 | 40.1 | PMHI | English | SG | (Mental Health) construct validity, criterion validity, internal consistency | X | Very good |
| Psychometric properties of the 8-item Morisky Medication Adherence Scale in patients taking warfarin | F + M | 151 | 56 | MMAS | English, Chinese | SG | internal consistency, construct validity, criterion validity | ✔️ | Adequate |
| Psychometric properties of the Chinese version of Problem Areas In Diabetes Scale (Sg-Paid-C) among high-risk polypharmacy patients with uncontrolled type 2 diabetes In Singapore | F + M | 211 | 61.7 | Sg-Paid-C | Chinese | SG | (DM) construct validity, internal consistency | X | Very good |
| Psychometric Properties of the Chinese Version of the Acceptance of Chronic Health Conditions (Stoma) Scale for Patients With Stoma | F + M | 100 | 64 | ACHC (Stoma) scale | Chinese | SG | (Colorectal Cancer) content validity, internal consistency, test-retest reliability, construct validity | X | Very good |
| Psychometric Properties of the Functional Assessment of Cancer Therapy-Neutropenia in Asian Cancer Patients With Chemotherapy-Induced Neutropenia | F + M | 276 | 54.6 | FACT-N | English, Chinese | SG | (Chemotherapy-induced Neutropenia) construct validity^m^, internal consistency | ✔️ | Very good |
| Psychometric properties of the positive mental health instrument among people with mental disorders: a cross sectional study | F + M | 360 | 39.2 | PMHI | English | SG | (Schizophrenia, Depression or Anxiety Spectrum Disorders) construct validity, internal consistency, criterion validity | ✔️ | Adequate |
| Psychometric Properties of the Problem Areas in Diabetes (PAID) Instrument in Singapore | F + M | 203 | 45 | PAID | English | SG | (DM) construct validity, criterion validity, internal consistency | X | Very good |
| Psychometric properties of the short Warwick Edinburgh mental well being scale (SWEMWBS) in service users with schizophrenia, depression and anxiety spectrum disorders | F + M | 350 | 39.1 | SWEMWBS | English | SG | (Schizophrenia, Depression and Anxiety Spectrum Disorders) construct validity, internal consistency | X | Adequate |
| Psychometric properties of the thyroid-specific quality of life questionnaire ThyPRO in Singaporean patients with Graves’ disease | F + M | 47 | 38.5 | ThyPRO questionnaire | English | SG | (Thyroid/Grave’s Disease) internal consistency, content validity, construct validity, responsiveness | X | Doubtful |
| Psychometric properties of the World Health Organization WHOQOL-AGE Scale in Singapore | F + M | 593 | 67.19 | WHOQOL-AGE | English | SG | construct validity, internal consistency | ✔️ | Very good |
| Psychometric properties of the World Health Organization WHOQOL-BREF Quality of Life assessment in Singapore | F + M | 3400 | 45.53 | WHOQOL-BREF | English | SG | construct validity, internal consistency, criterion validity | X | Very good |
| Psychometric testing of the Functional Assessment of Cancer Therapy/Gynecologic Oncology Group—Neurotoxicity (FACT/GOG-Ntx) subscale in a longitudinal study of cancer patients treated with chemotherapy | F + M | 343 | 55.2 | FACT/GOG-Ntx | English, Chinese | SG + M | (Cancer) internal consistency, construct validity, responsiveness | X | Adequate |
| Psychometric validation of the Hypoglycemia Fear Survey-II (HFS-II) in Singapore | F + M | 150 | 45.9 | HFS-II | English, Chinese | SG | (DM) content validity, criterion validity, construct validity, internal consistency, test-retest reliability | ✔️ | Doubtful |
| Quick-FLIC: validation of a short questionnaire for assessing quality of life of cancer patients | F + M | 190 | 55 | Quick-FLIC | Chinese | SG | (Cancer) internal consistency, construct validity, test-retest reliability | ✔️ | Adequate |
| Quick-FLIC: validation of a short questionnaire for assessing quality of life of cancer patients | F + M | 190 | 55 | Quick-FLIC | Chinese | SG | (Cancer) construct validity, criterion validity, internal consistency, test–retest reliability, responsiveness | X | Very good |
| Reliability and validity of the English (Singapore) and Chinese (Singapore) versions of the Short-Form 36 version 2 in a multi-ethnic Urban Asian population in Singapore | F + M | 4917 | 49.95 | SF-36v2 | English, Chinese | SG | internal consistency, construct validity | ✔️ | Adequate |
| Reliability and validity of the English-, Chinese- and Malay-language versions of the World Health Organization quality of life (WHOQOL-BREF) questionnaire in Singapore | F + M | 1316 | 51.9 | WHOQOL-BREF | English, Chinese, Malay | SG | construct validity, internal consistency, test-retest reliability | ✔️ | Very good |
| Screening for cognitive symptoms in dialysis patients with an extended version of Kidney Disease Quality of Life Cognitive Function subscale (KDQOL-CF): a validation study | F + M | 268 | 59.87 | KDQOL-CF | English, Chinese | SG | (HD) Construct validity, internal consistency | X | Very good |
| Screening for depressive disorders: Validation of the Patient Health Questionnaire for Adolescents (PHQ-A) in a population-based multi-ethnic Asian sample | F + M | 323 | 15.1 | PHQ-A | English | SG | (Depressive Disorders) internal consistency, construct validity | ✔️ | Very good |
| Screening for depressive symptoms: validation of the center for epidemiologic studies depression scale (CES-D) in a multiethnic group of patients with diabetes in Singapore | F + M | 522 | 54.5 | CES-D | English, Chinese, Malay | SG | (DM) inter-rater reliability, internal consistency | ✔️ |  |
| Screening for major and minor depression in a multiethnic sample of Asian primary care patients: a comparison of the nine-item Patient Health Questionnaire (PHQ-9) and the 16-item Quick Inventory of Depressive Symptomatology - Self-Report (QIDS-SR16 ) | F + M | 400 | 36.1 | PHQ-9, QIDS-SR16 | English | SG | (Depression) internal consistency, construct validity, criterion validity | ✔️ | Very good |
| Testing reliability and validity of oral impacts on daily performances for Chinese-speaking elderly Singaporeans | F + M | 202 | 75 | OIDP | Chinese | SG | test-retest reliability, internal consistency | ✔️ |  |
| Test-retest Reliability for HAQ-DI and SF-36 PF for the Measurement of Physical Function in Psoriatic Arthritis | F + M | 238 | 54 | HAQ-DI, SF-36 | English | SG + M | (Psoriatic Arthritis) test-retest reliability | X |  |
| Test-retest reliability of the Mandarin versions of the Hypertension Self-Care Profile instrument | F + M | 153 | 59 | HTN-SCP | Chinese | SG | (HTN) test-retest reliability, internal consistency | X |  |
| The Distress Thermometer as an ultra-short screening tool: a first validation study for mixed-cancer outpatients in Singapore | F + M | 105 | majority 51–60 | DT | English | SG | (Cancer) criterion validity, construct validity | X | Adequate |
| The English and Chinese versions of the five-level EuroQoL Group's five-dimension questionnaire (EQ-5D) were valid and reliable and provided comparable scores in Asian breast cancer patients | F | 269 | 52.65 | EQ-5D | English, Chinese | SG | (Breast Cancer) test-retest reliability, construct validity, responsiveness | ✔️ | Adequate |
| The equivalence and difference between the English and Chinese versions of two major, cancer-specific, health-related quality-of-life questionnaires | F + M | 1136 | 51.6 | FACT-G, EORTC QLQ-C30 | English, Chinese | SG | (Cancer) construct validity^m^ | ✔️ | Very good |
| The equivalence of English and Chinese SF-36 versions in bilingual Singapore Chinese | F + M | 168 | 35.7 | SF-36 | English, Chinese | SG | internal consistency, construct validity^m^ | X | Adequate |
| The European Organization for Research and Treatment of Cancer Quality of Life Questionnaire (EORTC QLQ-C30): Validation of English version in Singapore | F + M | 57 | 43 | EORTC QLQ-C30 | English | SG | (Cancer) internal consistency, construct validity | X | Adequate |
| The oxford knee score minimal clinically important difference for revision total knee arthroplasty | F + M | 191 | 67.9 | OKS | English | SG | (TKA) interpretability | X |  |
| The positive mental health instrument: development and validation of a culturally relevant scale in a multi-ethnic Asian population | F + M | 2088 | 41 | PMHI | English | SG | (Mental Health) internal consistency, criterion validity, interpretability | ✔️ |  |
| The Recovering Quality of Life 10-item (ReQoL-10) scale in a first-episode psychosis population: Validation and implications for patient-reported outcome measures (PROMs) | F + M | 300 | 27.2 | ReQoL-10 scale | English | SG | (Psychosis) internal consistency, construct validity | X | Adequate |
| The reliability, validity and sensitivity to change of the Chinese version of SF-36 in oriental patients with rheumatoid arthritis | F + M | 401 | 57 | SF-36 | Chinese | SG | (Rheumatoid Arthritis) test-retest reliability, internal consistency, responsiveness, construct validity | X | Adequate |
| The Rheumatology Attitudes Index and its Helplessness subscale are valid and reliable measures of learned helplessness in Asian SLE patients | F + M | 120 | 28 | RAI | English | SG | (SLE) internal consistency, test-retest reliability, construct validity | X | Very good |
| The Short Form 36 English and Chinese versions were equivalent in a multiethnic Asian population | F + M | 4,973 | 49.5 | SF-36v2 | English, Chinese | SG | construct validity^m^ | ✔️ | Adequate |
| The Singaporean English and Chinese versions of the EQ-5D achieved measurement equivalence in cancer patients | F + M | 771 | 52 | EQ-5D | English, Chinese | SG | (Cancer) construct validity^m^, responsiveness, test-retest reliability | ✔️ | Very good |
| The validity of pain intensity measures: What do the NRS, VAS, VRS, and FPS-R measure? | F + M | 101 | 48.3 | NRS, VAS, VRS, FPS-R | English | SG | internal consistency, criterion validity, test-retest reliability | X |  |
| Translation and cultural adaption of the Chronic Liver Disease Questionnaire for the Mandarin-speaking Chinese population in Singapore through cognitive debriefing | F + M | 18 | 49 | CLDQ | Chinese | SG | (Chronic Liver Disease) internal consistency, test-retest reliability | ✔️ |  |
| Translation and Validation of the 10-Item FAMCARE Scale to Assess Satisfaction of Family Caregivers With Care Given to Cancer Patients | F + M | 259 | 51.4 | 10-Item FAMCARE Scale | English, Chinese | SG | (Cancer) test-retest reliability, internal consistency, construct validity | ✔️ | Very good |
| Two valid and reliable short forms of the Singapore caregiver quality of life scale were developed: SCQOLS-10 and SCQOLS-15 | F + M | 612 | 48 | SCQOLS | English, Chinese | SG | (Cancer) test-retest reliability, internal consistency, content validity | ✔️ |  |
| Use of patient health questionnaires (PHQ-9, PHQ-2 & PHQ-1) for depression screening in Singapore primary care | F + M | 400 | 36.1 | PHQ-1, PHQ-2, PHQ-9 | English | SG | (Depression) criterion validity, internal consistency | ✔️ |  |
| Usefulness of the Audit of Diabetes-Dependent Quality-of-Life (ADDQoL) questionnaire in patients with diabetes in a multi-ethnic Asian country | F + M | 152 | 52 | ADDQoL | English | SG | (DM) internal consistency, content validity, construct validity | X | Adequate |
| Utility of EQ-5D to assess patients undergoing cataract surgery | F + M | 216 | 66 | EQ-5D, VF-14 | English, Chinese | SG | (Cataract Surgery) construct validity, responsiveness | ✔️ | Very good |
| Validation and interval scale transformation of the Western Ontario and McMaster Universities Osteoarthritis Index (WOMAC) in patients undergoing knee arthroplasty, using the Rasch model | F + M | 1136 | 65.9 | WOMAC | English, Chinese | SG | (TKR / TKA) construct validity, internal consistency, responsiveness | X | Adequate |
| Validation of a New Diabetic Retinopathy Knowledge and Attitudes Questionnaire in People with Diabetic Retinopathy and Diabetic Macular Edema | F + M | 36 | 60.1 | DRKA | English, Chinese | SG | (DM) construct validity, criterion validity, temporal (test-retest) reliability | ✔️ | Doubtful |
| Validation of a questionnaire measuring patient knowledge of atrial fibrillation in an Asian cohort | F + M | 165 | 68.7 | AF knowledge, attitude and perceptions questionnaire | English, Chinese | SG | (AF) content validity, construct validity, Internal consistency | ✔️ | Doubtful |
| Validation of Chinese Western Ontario and McMaster Universities Osteoarthritis Index (WOMAC) in patients scheduled for total knee replacement | F + M | 258 | 66.6 | WOMAC | English, Chinese | SG | (TKR / TKA) construct validity, internal consistency, test–retest reliability | ✔️ | Adequate |
| Validation of EORTC QLQ-C30 and QLQ-BR23 questionnaires in the measurement of quality of life of breast cancer patients in Singapore | F | 170 | 54 | EORTC QLQ-C30, QLQ-BR23 | English, Chinese, Malay, Tamil | SG | (Breast Cancer) internal consistency, criterion validity | X |  |
| Validation of PDQ-8 as an independent instrument in English and Chinese | F + M | 183 | 61 | PDQ-8 | English, Chinese | SG | (Parkinson's Disease) internal consistency, construct validity | X | Very good |
| Validation of screening questionnaires for evaluation of knee osteoarthritis prevalence in the general population of Singapore | F + M | 146 | 60.4 | screening questionnaires for identification of symptomatic KOA | English, Chinese | SG | (KOA) validity (content validity, criterion validity, and construct validity) | X | Doubtful |
| Validation of the Chinese Manchester foot pain and disability index (C-MFPDI) among patients with inflammatory arthritis | F + M | 100 | 52.9 | C-MFPDI | Chinese | SG | (Inflammatory Arthritis) internal consistency, criterion validity, content validity, test-retest reliability | X |  |
| Validation of the Chinese SF-36 for quality of life assessment in patients with systemic lupus erythematosus | F + M | 69 | 32.1 | SF-36 | Chinese | SG | (SLE) construct validity, internal consistency, test-retest reliability | X | Adequate |
| Validation of the emotion thermometers and hospital anxiety and depression scales in Singapore: Screening cancer patients for distress, anxiety and depression | F + M | 315 | 59.4 | ET, HADS | English | SG | (Cancer) criterion validity, internal consistency | X |  |
| Validation of the English and Chinese versions of the Quick-FLIC quality of life questionnaire | F + M | 548 | 49.4 | Quick-FLIC | English, Chinese | SG | (Cancer) criterion validity, test-retest reliability, responsiveness, internal consistency, construct validity | ✔️ | Adequate |
| Validation of the English and simplified Mandarin versions of the Fear of Progression Questionnaire-Short form in Chinese cancer survivors | F + M | 341 | 55.32 | FoP-Q-SF | English, Chinese | SG | (Cancer) test-retest reliability, internal consistency, criterion validity | X |  |
| Validation of the English version of the Kidney Disease Quality of Life questionnaire (KDQOL-36) in haemodialysis patients in Singapore | F + M | 394 | 52.4 | KDQOL-36 | English | SG | (HD) internal consistency, construct validity, criterion validity | ✔️ | Very good |
| Validation of the English version of the KINDL generic children's health-related quality of life instrument for an Asian population--results from a pilot test | F + M | 69 | 10.7 | KINDL-Kid and KINDL-Kiddo | English | SG | (DM) construct validity, internal consistency | ✔️ | Doubtful |
| Validation of the functional assessment of cancer therapy-gastric module for the Chinese population | F + M | 67 | 67.4 | FACT-Ga | English, Chinese | SG | (Gastric Cancer) construct validity, internal consistency | ✔️ | Adequate |
| Validation of the kidney disease quality of life-short form: a cross-sectional study of a dialysis-targeted health measure in Singapore | F + M | 980 | 56 | KDQOL-SF | English, Chinese, Malay | SG | (HD) construct validity, internal consistency | X | Adequate |
| Validation of the medical outcomes study family and marital functioning measures in SLE patients in Singapore | F + M | 118 | 28 | FMM and MFM | English | SG | (SLE) internal consistency, construct validity | X | Very good |
| Validation of the mental health continuum-short form: The bifactor model of emotional, social, and psychological well-being | F + M | 299 | 24.26 | MHC-SF | English | SG + M | (Mental Health) construct validity, internal consistency, criterion validity | X | Very good |
| Validation of the Recap of Atopic Eczema (RECAP) Measurement Instrument for Eczema Control in Adult Patients in an Asian Clinical Setting | F + M | 260 | 30.36 | RECAP | English | SG | (Atopic Eczema) construct validity, reliability (internal consistency and measurement error) | X | Very good |
| Validation of the scleroderma health assessment questionnaire and quality of life in English and Chinese-speaking patients with systemic sclerosis | F + M | 49 | 55 | S-HAQ, SSc-QoL | English, Chinese | SG | (Systemic Sclerosis) Test-retest reliability, construct validity | ✔️ | Doubtful |
| Validation of the Stroke and Aphasia Quality of Life Scale in a multicultural population | F + M | 94 | 63.7 | SAQOL-39 g/ SAQOL-CSg | English, Chinese | SG | (Stroke) Internal consistency, Test–retest reliability, construct validity | ✔️ |  |
| Validation of the UCLA Scleroderma Clinical Trial Consortium Gastrointestinal Tract Instrument 2.0 in English- and Chinese-speaking patients in a multi-ethnic Singapore systemic sclerosis cohort | F + M | 220 | 51 | GIT 2.0 | English, Chinese | SG | (Systemic Sclerosis) construct validity, test-retest reliability, internal consistency | X | Adequate |
| Validation of the Western Ontario and Mcmaster University osteoarthritis index in Asians with osteoarthritis in Singapore | F + M | 66 | 54.9 | WOMAC | English | SG | (OA) internal consistency, construct validity, test-retest reliability | ✔️ | Adequate |
| Validity and reliability of EQ-5D-5L among patients with axial spondyloarthritis in Singapore | F + M | 118 | 35 | EQ-5D-5L | English | SG | (Axial Spondyloarthritis) construct validity, internal consistency, test-retest reliability, Interpretability | X | Very good |
| Validity and Reliability of the American Orthopaedic Foot and Ankle Society Score for the English-Literate Singapore Population With Hallux Valgus | F + M | 121 | 58.3 | AOFAS | English | SG | (Hallux Valgus) construct validity, internal consistency, responsiveness | X | Adequate |
| Validity and reliability of the Ankylosing Spondylitis Disease Activity Score with C-reactive protein (ASDAS-CRP) and Bath Ankylosing Spondylitis Disease Activity Index (BASDAI) in patients with axial spondyloarthritis (axSpA) in Singapore | F + M | 280 | 39 | ASDAS-CRP, BASDAI | English | SG | (Axial Spondyloarthritis) Internal consistency, Construct validity | X | Very good |
| Validity and reliability of the Assessment of Spondyloarthritis International Society Health Index in English-speaking patients with axial spondyloarthritis in Singapore | F + M | 108 | 37 | ASAS HI | English | SG | (Axial Spondyloarthritis) Construct validity, content validity, internal consistency, test-retest reliability, measurement error, interpretability | ✔️ | Very good |
| Validity and reliability of the Chinese (Singapore) version of the Parkinson's disease questionnaire (PDQ-39) | F + M | 63 | 65 | PDQ-39 | Chinese | SG | (Parkinson’s Disease) construct validity, content validity, test-retest reliability, internal consistency | ✔️ | Doubtful |
| Validity and reliability of the English and translated Chinese versions of the Integrated Palliative care Outcome Scale (IPOS) in Singapore | F + M | 220 | 61.3 | IPOS | English, Chinese | SG | (Palliative Care) construct validity, inter-rater reliability, internal consistency, test-retest reliability | ✔️ | Very good |
| Validity and reliability of the EQ-5D self-report questionnaire in Chinese-speaking patients with rheumatic diseases in Singapore | F + M | 48 | 56.4 | EQ-5D | Chinese | SG | (Rheumatic Disease) construct validity, test-retest reliability | ✔️ | Doubtful |
| Validity and reliability of the EQ-5D self-report questionnaire in English-speaking Asian patients with rheumatic diseases in Singapore | F + M | 66 | 44.3 | EQ-5D | English | SG | (Rheumatic Disease) construct validity, test-retest reliability | ✔️ | Adequate |
| Validity and Reliability of the European Foot and Ankle Society (EFAS) Score in Patients With Hallux Valgus in Singapore | F + M | 121 | 58.3 | EFAS | English | SG | (Hallux Valgus) internal consistency, construct validity, interpretability | X | Adequate |
| Validity and reliability of the Gout Impact Scale in a multi-ethnic Asian population | F + M | 267 | 52.2 | GIS | English | SG | (Gout) construct validity, internal consistency | ✔️ | Very good |
| Validity and reliability of the Health Assessment Questionnaire among patients with spondyloarthritis in Singapore | F + M | 196 | 36 | HAQ | English | SG | (Axial Spondyloarthritis) construct validity, internal consistency | X | Very good |
| Validity and Reliability of the Integrated Palliative Care Outcome Scale in Asian Heart Failure Patients | F + M | 91 | 56.5 | IPOS | English | SG | (Heart Failure) internal consistency, test–retest reliability, construct validity, Inter-rater reliability | ✔️ | Adequate |
| Validity and reliability of the MD Anderson dysphagia inventory in English and Chinese in head and neck cancer patients | F + M | 66 | 61.5 | MDADI | English, Chinese | SG | (Head and Neck Cancer) Test–retest reliability, Internal consistency, Criterion validity, Construct validity, content validity | X | Adequate |
| Validity and reliability of the PDQ-39 and the PDQ-8 in English-speaking Parkinson's disease patients in Singapore | F + M | 88 | 63.1 | PDQ-39, PDQ-8 | English | SG | (Parkinson's Disease) construct validity, internal consistency, Test–retest reliability | ✔️ | Adequate |
| Validity and reliability of the Short Form 36 Health Surveys (SF-36) among patients with spondyloarthritis in Singapore | F + M | 196 | 36 | SF-36 | English | SG | (Axial Spondyloarthritis) internal consistency, construct validity | X | Very good |
| Validity and reliability of the ten-item Connor-Davidson Resilience Scale (CD-RISC10) instrument in patients with axial spondyloarthritis (axSpA) in Singapore | F + M | 108 | 37 | CD-RISC10 | English | SG | (Axial Spondyloarthritis) content validity, construct validity, test-retest reliability, internal consistency, interpretability, measurement error | ✔️ | Adequate |
| Validity and reliability of Work Productivity and Activity Impairment among patients with axial spondyloarthritis in Singapore | F + M | 168 | 41 | WPAI | English | SG | (Axial Spondyloarthritis) content validity, construct validity, test-retest reliability, Measurement error, Interpretability | ✔️ | Very good |
| Validity of a Revised Short Form-12 Health Survey Version 2 in Different Ethnic Populations | F + M | 7188 | 49.4 | SF-12v2 | English, Chinese | SG | content validity, criterion validity, construct validity | ✔️ | Adequate |
| Validity of EuroQOL-5D, time trade-off, and standard gamble for age-related macular degeneration in the Singapore population | F + M | 338 | 68.1 | EQ-5D | English, Chinese, Malay | SG | (ARMD) construct validity | ✔️ | Doubtful |
| Validity of the patient health questionnaire 9-item in autistic youths: a pilot study | F + M | 101 | 14.6 | PHQ-9 | English | SG | (ASD) internal consistency, construct validity | X | Adequate |
| What is the test-retest reliability of the Malay version of the Hypertension Self-Care Profile self efficacy assessment tool? A validation study in primary care | F + M | 145 | 58 | HTN-SCP | Malay | SG | (HTN) internal consistency, test-retest reliability | X |  |

Abbreviations : shortened version of the Western Ontario and McMaster Universities Osteoarthritis Index function scale (Modified ShortMAC-F), Western Ontario and McMaster Universities Osteoarthritis Index (WOMAC), Knee injury and Osteoarthritis Outcome Score (KOOS), Kansas City Cardiomyopathy Questionnaire (KCCQ), Stroke and Aphasia Quality of Life Scale (SAQOL-39 g) and its Mandarin adaptation (SAQOL-CSg), Functional Assessment of Cancer Therapy-Breast (FACT-B), Integrated Palliative care Outcome Scale (IPOS), Problem Areas in Diabetes (PAID), Diabetes Health Profile-18 (DHP-18), Diabetic Retinopathy Knowledge and Attitudes (DRKA), Diabetes-Related Nutrition Knowledge (DRNK), Audit of Diabetes-Dependent Quality-of-Life (ADDQoL), Hypoglycemia Fear Survey-II (HFS-II), Singapore Caregiver Quality of Life Scale - 10-item / 15-item (SCQOLS-10/SCQOLS-15), Singapore Caregiver Quality of Life Scale - Dementia (SCQOLS-D), Family Satisfaction with Care Scale (10-Item FAMCARE scale), Quality of Life (QoL), five-level EuroQoL Group's five-dimension questionnaire (EQ-5D-5L/EQ-5D), five-level EuroQoL Group's five-dimension questionnaire descriptive system (EQ-5D-Y DS), 36-Item Short Form Health Survey / 36-Item Short Form Health Survey version 2 (SF-36/SF-36v2), Health Utilities Index Mark 3 (HUI3), Pain Catastrophizing Scale (PCS), World Health Organization Quality of Life-BREF (WHOQOL-BREF), Health-Related Quality of Life (HRQoL), Beck Anxiety Inventory (BAI), Hospital Anxiety and Depression Scales (HADS), Distress Thermometer (DT), Lower Extremity Functional Scale (LEFS), Morisky Medication Adherence Scale (MMAS), Health Assessment Questionnaire (HAQ), Health Assessment Questionnaire-Disability Index (HAQ-DI), Scleroderma Health Assessment Questionnaire (S-HAQ), Oral Impacts on Daily Performances (OIDP), Numerical Rating Scale (NRS), Visual Analogue Scale (VAS), Verbal Rating Scale (VRS), Faces Pain Scale-Revised (FPS-R), Quick Version of the Functional Living Index-Cancer (Quick-FLIC), MD Anderson Dysphagia Inventory (MDADI), Connor-Davidson Resilience Scale (CD-RISC10), Singapore Mental Wellbeing (SMWEB), Medical Outcomes Study Family and Marital Functioning Measures (FMM and MFM), nine-item Patient Health Questionnaire (PHQ-9), one-item Patient Health Questionnaire (PHQ-1), two-item Patient Health Questionnaire (PHQ-2), Adult Sedentary Behaviour Questionnaire (ASBQ), 16-item Quick Inventory of Depressive Symptomatology – Self-Report (QIDS-SR16), Multidimensional Fatigue Syndrome Inventory- Short Form (MFSI-SF), Short Form 6-Dimension (SF-6D), Work Productivity and Activity Impairment (WPAI), European Organization for Research and Treatment of Cancer Quality of Life Questionnaire (EORTC QLQ-C30), Quality of Life Questionnaire – Breast Cancer Specific Module (QLQ-BR23), Short Form-12 version 2 (SF-12v2), Emotion Thermometer (ET), Instrumental Activities of Daily Living (IADL), Center for Epidemiologic Studies Depression Scale (CES-D), 8-item Altarum Consumer Engagement Measure™ (ACE Measure), Rapid Positive Mental Health Instrument (R-PMHI), Control, Autonomy, Self-realization, Pleasure Quality of Life scale (CASP‑11‑SG scale), Functional Assessment of Cancer Therapy - General (FACT-G), Functional Assessment of Cancer Therapy: Cognitive Function (FACT-Cog), Childhood Asthma Questionnaire (CAQ-B), Paediatric Asthma Quality of Life Questionnaire (PAQLQ), Kidney Disease Quality of Life Short Form (KDQOL-SF/KDQOL-36), Kidney Disease Quality of Life Cognitive Function subscale (KDQOL-CF), Dermatology Social Comparison (DSC), Recap of Atopic Eczema Patient-Reported Outcomes (RECAP), Systemic Lupus Erythematosus-Specific Quality-Of-Life instrument (SLEQOL), Systemic Lupus Erythematosus Quality of Life Questionnaire - Chinese version (SLEQOL-C), Hemifacial spasm-30 (HFS-30), Thyroid-specific quality of life questionnaire (ThyPRO questionnaire), Chronic Liver Disease Questionnaire (CLDQ), Chronic Liver Disease Questionnaire - Singapore-Mandarin version (CLDQ-SG), Hepatitis Quality of Life Questionnaire (HQLQ), 8-item Parkinson's Disease Questionnaire (PDQ-8), 39-item Parkinson's Disease Questionnaire (PDQ-39), Functional Assessment of Cancer Therapy-Gastric Module (FACT-Ga), European Foot and Ankle Society (EFAS), Assessment of Spondyloarthritis International Society Health Index (ASAS HI), Rheumatoid Arthritis Impact of Disease (RAID), Bath Ankylosing Spondylitis Disease Activity Index (BASDAI), Psoriatic Arthritis Quality of Life (PsAQoL), Gout Impact Scale (GIS), Ankylosing Spondylitis quality of life (ASQoL), Rheumatology Attitudes Index (RAI), American Orthopaedic Foot and Ankle Society (AOFAS), Chinese Manchester foot pain and disability index (C-MFPDI), Knee Osteoarthritis (KOA), Osteoarthritis (OA), Impact of Vision Impairment (IVI), Visual Function Index-11 (VF-11), Visual Function Index-14 (VF-14), Hypertension Self-Care Profile (HTN-SCP), Atrial fibrillation (AF), Singapore (SG), Multinational (M), Diabetes mellitus (DM), Hypertension (HTN), Total Knee Replacement /Total Knee Arthroplasty (TKR/TKA), Systemic lupus erythematosus (SLE), Age-Related Macular Degeneration (ARMD), haemodialysis (HD), Inpatient Dignity Scale (IPDS), short multidimensional positive mental health instrument (SMPMHI), Functional Living Index for Cancer (FLIC), End stage renal disease (ESRD), Quality-of-Life Measure for Hidradenitis Suppurativa (HS-QoL), Hidradenitis Suppurativa (HS), Functional Assessment of Cancer Therapy/Gynecologic Oncology Group—Neurotoxicity (FACT/GOG-Ntx), Oxford Knee Score (OKS), Arthroscopic Rotator Cuff Repair (Arthroscopic RCR), Oxford Shoulder Instability Score (OSIS), Ankylosing Spondylitis Disease Activity Score with C-reactive protein (ASDAS-CRP), Oxford Hip Score (OHS), Total Hip Arthroplasty (THA), Asian Children Depression Scale (ACDS), Asian Adolescent Depression Scale (AADS), Geriatric Depression Screening Scale (GDS-15), Global Physical Activity Questionnaire (GPAQ), Short-Form of the McGill Pain Questionnaire (MPQ-SF), positive mental health instrument (PMHI), U.S. National Cancer Institute’s
Patient-Reported Outcomes version of the Common Terminology Criteria for Adverse Events (PRO-CTCAE), Recovering Quality of Life 10-item (ReQoL-10) scale, Systemic Sclerosis Quality of Life scale (SSc-QoL), 13-items World Health Organization Quality of Life Assessment-Older Adults Module (WHOQOL-AGE), Mental Health Continuum-Short Form (MHC-SF), Fear of Progression Questionnaire – Short Form (FoP-Q-SF), Schizophrenia Quality of Life Scale (SQLS), Acceptance of Chronic Health Conditions (ACHC), Functional Assessment of Cancer Therapy-Neutropenia (FACT-N), Short Warwick Edinburgh Mental Well-Being Scale (SWEMWBS), Patient Health Questionnaire for Adolescents (PHQ-A), Gastrointestinal Tract Instrument (GIT), Autism Spectrum Disorder (ASD), Multiple sclerosis international quality of life questionnaire (MusiQoL), Singapore Thyroid Eye Disease Quality of Life questionnaire (STED-QoL), measurement equivalence/invariance classified as construct validity (construct validity^m^)
