# Supplementary material for: A repository of Singapore validated PROMS: a scoping review
Source: J Patient Rep Outcomes. 2026 Feb 23;10:48. doi: 10.1186/s41687-026-01005-4 (PMC13031579; doi:10.1186/s41687-026-01005-4)
Supplement: Supplementary file 3 — Supplementary Material 3 [file 41687_2026_1005_MOESM3_ESM.docx]

| **(Disease conditions including 19 VDC (*) / PROMS involved)/Psychometric properties** | **Language (s)** | **Indication** | **Psychometric properties** | **Paper(s) link** |
| --- | --- | --- | --- | --- |
|  |  |  |  |  |
| **Total Knee Replacement / Total Knee Arthroplasty*** |  |  |  |  |
| Modified ShortMAC-F | English | TKA | internal consistency, construct validity, interpretability | <https://doi.org/10.1016/j.apmr.2024.05.005> |
| WOMAC | English, Chinese | TKA, OA | internal consistency, test-retest reliability, construct validity, responsiveness, interpretability | [https://doi.org/10.1007/s11136-008-9340-7  https://doi.org/10.1016/j.ocarto.2022.100322](https://doi.org/10.1007/s11136-008-9340-7)  <https://doi.org/10.1016/j.arth.2020.04.034>   <https://doi.org/10.1053/joca.2000.0410> |
| Lequesne Algofunctional Index of knee | English, Chinese | TKR | internal consistency, test-retest reliability, construct validity | <https://doi.org/10.1016/j.joca.2006.06.013> |
| KOOS | English, Chinese | TKR | internal consistency, test-retest reliability, construct validity | <https://doi.org/10.1016/j.joca.2006.05.005> |
| OKS | English, Chinese | TKA | internal consistency, construct validity, interpretability | <https://doi.org/10.1016/j.knee.2021.08.020><https://pubmed.ncbi.nlm.nih.gov/17408984/> |
| **Total Hip Replacement / Total Hip Arthroplasty*** |  |  |  |  |
| OHS | English | THA | internal consistency, test-retest reliability, interpretability | <https://doi.org/10.1016/j.arth.2020.04.034> |
| **Cataract surgery*** |  |  |  |  |
| NIL | | | | |
| **Laparoscopic cholecystectomy*** |  |  |  |  |
| NIL | | | | |
| **Hysterectomy*** |  |  |  |  |
| NIL | | | | |
| **Coronary artery bypass graft*** |  |  |  |  |
| NIL | | | | |
| **Acute myocardial infarction*** |  |  |  |  |
| NIL | | | | |
| **Congestive heart failure*** |  |  |  |  |
| KCCQ | English | Heart Failure | internal consistency, construct validity | 10.1002/ehf2.12950 |
| **Ischemic stroke*** |  |  |  |  |
| SAQOL-39 g / SAQOL-CSg | English, Chinese | Stroke | internal consistency, test-retest reliability, construct validity | [https://doi.org//10.3109/09638288.2016.1138551](https://doi.org/10.3109/09638288.2016.1138551) |
| **Hernia repair*** |  |  |  |  |
| NIL | | | | |
| **Caesarean section*** |  |  |  |  |
| NIL | | | | |
| **Tonsillectomy*** |  |  |  |  |
| NIL | | | | |
| **Hemorrhoidectomy*** |  |  |  |  |
| NIL | | | | |
| **Pneumonia*** |  |  |  |  |
| NIL | | | | |
| **Colorectal resection*** |  |  |  |  |
| ACHC (Stoma) scale | Chinese | Colorectal cancer | Internal consistency, test-retest reliability, content validity, construct validity | [10.1097/NCC.0000000000000411](https://doi-org.libproxy1.nus.edu.sg/10.1097/ncc.0000000000000411) |
| **Spinal fusion*** |  |  |  |  |
| NIL | | | | |
| **Breast cancer*** |  |  |  |  |
| QLQ-BR23 | English, Chinese, Malay, Tamil | Breast Cancer | internal consistency, criterion validity | <https://doi.org/10.4103/2347-5625.135817> |
| FACT-B | English, Chinese | Breast Cancer | internal consistency, test-retest reliability, construct validity, responsiveness | [https://doi.org/10.1007/s11136-012-0291-7  https://doi.org/10.1007/s10549-011-1764-z](https://doi.org/10.1007/s11136-012-0291-7) |
| **End of life*** |  |  |  |  |
| IPOS | English, Chinese | Palliative Care, Heart Failure | internal consistency, test-retest reliability, inter-rater reliability, construct validity | [https://doi.org//10.1089/pmr.2022.0029](https://www-liebertpub-com.libproxy1.nus.edu.sg/doi/10.1089/pmr.2022.0029)  [https://doi.org//10.1186/s12904-021-00737-y](https://bmcpalliatcare-biomedcentral-com.libproxy1.nus.edu.sg/articles/10.1186/s12904-021-00737-y) |
| **Diabetes mellitus*** |  |  |  |  |
| PAID/Sg-Paid-C | English, Chinese | DM | internal consistency, criterion validity, construct validity | <https://doi.org/10.1111/jdi.12556> 10.1371/journal.pone.0136759 |
| DHP-18 | English | DM | internal consistency, construct validity | <https://pubmed.ncbi.nlm.nih.gov/27748785/> |
| DRKA | English, Chinese | DM | test-retest reliability, criterion validity, construct validity | <https://doi.org/10.1167/tvst.9.10.32> |
| DRNK | English | DM | internal consistency, content validity, construct validity, test-retest reliability | <https://doi.org/10.1111/1747-0080.12513> |
| ADDQoL | English, Chinese | DM | internal consistency, test-retest reliability, content validity, construct validity^m^, responsiveness | <https://doi.org/10.2165/00019053-200624070-00006>   <https://doi.org/10.2165/11313920-000000000-00000>   <https://doi.org/10.1016/j.vhri.2012.03.005> |
| HFS-II | English, Chinese | DM | content validity, criterion validity, construct validity, internal consistency, test-retest reliability | <https://doi.org/10.1136/bmjdrc-2016-000329> |
| **Caregiver-related** |  |  |  |  |
| SCQOLS-10/SCQOLS-15 | English, Chinese | Cancer, Heart Diseases | internal consistency, test-retest reliability, content validity, construct validity, criterion validity | <https://doi.org/10.1186/s12955-019-1108-y>   <https://doi.org/10.1016/j.jclinepi.2020.02.003>   [10.1007/s40271-023-00634-x](https://doi-org.libproxy1.nus.edu.sg/10.1007/s40271-023-00634-x) |
| SCQOLS-D | English | Dementia | internal consistency, test-retest reliability, content validity, criterion validity, construct validity | <https://doi.org/10.1186/s41687-021-00330-0>   <https://doi.org/10.1186/s41687-020-00252-3> |
| 10-Item FAMCARE scale | English, Chinese | Cancer | test-retest reliability, internal consistency, construct validity | <https://doi.org/10.1016/j.jpainsymman.2019.07.018> |
| **Chronic constipation** |  |  |  |  |
| Constipation PROM that measures both symptom severity and constipation-related QoL | English | Chronic Constipation | content validity | <https://doi.org/10.1016/j.csbj.2023.09.034> |
| **General** |  |  |  |  |
| EQ-5D-5L/EQ-5D/EQ-5D-Y DS | English, Chinese, Malay, Tamil | Axial Spondyloarthritis, Rheumatic Disease, Cancer, Breast Cancer, ARMD, Parkinson's Disease, Cataract Surgery, DM | internal consistency, test-retest reliability, content validity, construct validity, responsiveness, interpretability, construct validity^m^ | <https://doi.org/10.5152/eurjrheum.2020.19043>  <https://pubmed.ncbi.nlm.nih.gov/14528528/>   <https://pubmed.ncbi.nlm.nih.gov/14626802/>   <https://pubmed.ncbi.nlm.nih.gov/32582906/>   <https://doi.org/10.1007/s11136-012-0291-7>   <https://pubmed.ncbi.nlm.nih.gov/17597964/>   <https://doi.org/10.1007/s11136-014-0864-8>   <https://doi.org/10.1186/1477-7525-1-7>   <https://pubmed.ncbi.nlm.nih.gov/19565103/>   <https://doi.org/10.1007/s00520-012-1512-x>   <https://doi.org/10.1016/j.jclinepi.2008.03.007>   <https://doi.org/10.1097/OPX.0000000000000004>   <https://doi.org/10.1023/a:1022063721237>   <https://doi.org/10.1038/eye.2011.218>   <https://doi.org/10.1186/s12955-024-02290-7>   https://doi.org/10.1186/s12955-015-0297-2 |
| SF-36/SF-36v2 | English, Chinese | Rheumatoid Arthritis, SLE, Axial Spondyloarthritis, Psoriatic Arthritis, THA | internal consistency, test-retest reliability, construct validity, construct validity^m^, responsiveness, interpretability | <https://pubmed.ncbi.nlm.nih.gov/23475690/>  <https://pubmed.ncbi.nlm.nih.gov/33858982/>   <https://pubmed.ncbi.nlm.nih.gov/11642688/>   <https://link.springer.com/article/10.1023/A:1015680029998>   <https://doi.org/10.1093/rheumatology/kel051>   <https://pubmed.ncbi.nlm.nih.gov/11199927/>   <https://pubmed.ncbi.nlm.nih.gov/27664139/>   <https://doi.org/10.1016/j.arth.2020.04.034>   <https://doi.org/10.1016/j.jclinepi.2012.12.016> |
| HUI3 | English, Chinese | Rheumatic Disease | Test-retest reliability, construct validity | <https://pubmed.ncbi.nlm.nih.gov/14528528/> |
| Self-report measure of the extent of and reasons for medication nonadherence | English, Chinese, Malay | DM | Interpretability, content validity, internal consistency, test–retest reliability, construct validity, measurement error | <https://pubmed.ncbi.nlm.nih.gov/31551654/> |
| PCS | English | TKR | internal consistency, construct validity | <https://doi.org/10.1007/s10067-020-05163-8> |
| WHOQOL-BREF/WHOQOL-AGE | English, Chinese, Malay |  | construct validity, internal consistency, test-retest reliability, criterion validity, construct validity^m^ | <https://hqlo.biomedcentral.com/articles/10.1186/s12955-019-1130-0>  <https://pubmed.ncbi.nlm.nih.gov/29355283/>   <https://doi.org/10.1007/s10433-024-00803-3>   <https://doi.org/10.1007/s11136-018-1947-8> |
| item bank to measure HRQoL | English, Chinese |  | internal consistency, content validity, criterion validity, construct validity | <https://hqlo.biomedcentral.com/articles/10.1186/s12955-019-1150-9>  <https://doi.org/10.1186/s12955-019-1255-1> |
| BAI | English, Chinese | Breast Cancer | internal consistency, criterion validity, construct validity^m^ | <https://pubmed.ncbi.nlm.nih.gov/27771785/> |
| HADS | English | Cancer | criterion validity, internal consistency | <https://pubmed.ncbi.nlm.nih.gov/24673756/> |
| DT | English | Cancer | criterion validity, construct validity | <https://doi.org/10.1016/j.comppsych.2014.01.008> |
| LEFS | English |  | internal consistency, measurement error, content validity, criterion validity, construct validity | <https://pubmed.ncbi.nlm.nih.gov/38389197/> |
| MMAS | English, Chinese |  | internal consistency, construct validity, criterion validity | <https://pubmed.ncbi.nlm.nih.gov/22836920/> |
| HAQ/HAQ-DI | English, Chinese | Psoriatic Arthritis, Axial Spondyloarthritis, Rheumatoid Arthritis | internal consistency, test-retest reliability, criterion validity, construct validity | <https://pubmed.ncbi.nlm.nih.gov/33858982/>  <https://onlinelibrary.wiley.com/doi/10.1111/1756-185X.12989>   <https://pubmed.ncbi.nlm.nih.gov/9733449/> |
| OIDP | Chinese |  | test-retest reliability, internal consistency | <https://pubmed.ncbi.nlm.nih.gov/25754187/> |
| NRS | English |  | internal consistency, criterion validity, test-retest reliability | <https://pubmed.ncbi.nlm.nih.gov/29794282/> |
| VAS | English | Arthroscopic RCR | internal consistency, criterion validity, test-retest reliability, interpretability | <https://pubmed.ncbi.nlm.nih.gov/29794282/><https://doi.org/10.1016/j.jisako.2024.05.003> |
| VRS | English |  | internal consistency, criterion validity, test-retest reliability | <https://pubmed.ncbi.nlm.nih.gov/29794282/> |
| FPS-R | English |  | internal consistency, criterion validity, test-retest reliability | <https://pubmed.ncbi.nlm.nih.gov/29794282/> |
| Quick-FLIC | English, Chinese | Cancer | internal consistency, test-retest reliability, criterion validity, construct validity, responsiveness | <https://pmc.ncbi.nlm.nih.gov/articles/PMC2361884/>  <https://doi.org/10.1038/sj.bjc.6601782>   <https://doi.org/10.1038/sj.bjc.6601782> |
| MDADI | English, Chinese | Head and Neck Cancer | Test–retest reliability, Internal consistency, Criterion validity, Construct validity, content validity | <https://pubmed.ncbi.nlm.nih.gov/32573112/> |
| CD-RISC10 | English | Axial Spondyloarthritis | content validity, construct validity, test-retest reliability, internal consistency, interpretability, measurement error | <https://link.springer.com/article/10.1007/s00296-018-4217-8> |
| FMM and MFM | English | SLE | internal consistency, construct validity | <https://pubmed.ncbi.nlm.nih.gov/10483028/> |
| PHQ-9/PHQ-1/PHQ-2 | English | Depression, ASD | internal consistency, criterion validity, construct validity | <https://onlinelibrary.wiley.com/doi/10.1111/appy.12101>  <https://cfps.org.sg/publications/the-singapore-family-physician/article/1223>   <https://doi.org/10.1186/s12888-021-03556-w> |
| ASBQ | English |  | construct validity, test-retest reliability | <https://doi.org/10.3390/ijerph15040739> |
| QIDS-SR16 | English | Depression | internal consistency, construct validity, criterion validity | <https://onlinelibrary.wiley.com/doi/10.1111/appy.12101> |
| MFSI-SF | English, Chinese | Breast Cancer, Lymphoma | internal consistency, construct validity^m^, responsiveness | <https://pubmed.ncbi.nlm.nih.gov/29351803/> |
| SF-6D | English, Chinese |  | construct validity^m^ | <https://doi.org/10.1016/S0149-2918(04)90186-5> |
| WPAI | English | Axial Spondyloarthritis | content validity, construct validity, test-retest reliability, Measurement error, Interpretability | <https://pubmed.ncbi.nlm.nih.gov/32020752/> |
| EORTC QLQ-C30 | English, Chinese, Malay, Tamil | Cancer, Breast Cancer | internal consistency, construct validity, criterion validity, construct validity^m^ | <https://pubmed.ncbi.nlm.nih.gov/15529310/>  10.1007/s11136-004-4782-z   <https://doi.org/10.4103/2347-5625.135817> |
| SF-12v2 | English, Chinese |  | content validity, criterion validity, construct validity | <https://pubmed.ncbi.nlm.nih.gov/27412055/> |
| ET | English | Cancer | criterion validity, internal consistency | <https://pubmed.ncbi.nlm.nih.gov/24673756/> |
| Questionnaire for the Assessment of Pruritus | English | Uremic Pruritus | content validity, criterion validity, construct validity, test-retest reliability | <https://pubmed.ncbi.nlm.nih.gov/11501646/> |
| Lawton and Brody's IADL | English, Chinese, Malay |  | internal consistency, construct validity | <https://doi.org/10.1093/gerona/61.7.726> |
| CES-D | English, Chinese, Malay | DM, Depression | internal consistency, inter-rater reliability, construct validity | <https://pubmed.ncbi.nlm.nih.gov/18023070/>  <https://pubmed.ncbi.nlm.nih.gov/18337303/> |
| ACE Measure | English |  | construct validity, criterion validity, internal consistency | <https://bmchealthservres.biomedcentral.com/articles/10.1186/s12913-021-07369-1> |
| CASP‑11‑SG scale | English, Chinese |  | internal consistency, construct validity | <https://pubmed.ncbi.nlm.nih.gov/36068420/> |
| FACT-G | English, Chinese | Cancer | construct validity, internal consistency, test-retest reliability, construct validity^m^ | <https://pubmed.ncbi.nlm.nih.gov/15529310/>  <https://www.annals.edu.sg/pdf/38VolNo3Mar2009/V38N3p225.pdf> |
| FACT-Cog | English, Chinese | Breast Cancer | internal consistency, test-retest reliability, criterion validity, construct validity, responsiveness, interpretability, construct validity^m^ | <https://pubmed.ncbi.nlm.nih.gov/24656406/><https://pubmed.ncbi.nlm.nih.gov/24041350/> |
| MPQ-SF | English | Uremic Pruritus | content validity, criterion validity, construct validity, test-retest reliability | <https://pubmed.ncbi.nlm.nih.gov/11501646/> |
| GPAQ | English |  | construct validity, test-retest reliability | <https://doi.org/10.3390/ijerph15040739> |
| GDS-15 | English, Chinese, Malay | Depression | criterion validity, test-retest reliability, inter-rater reliability, internal consistency | <https://pubmed.ncbi.nlm.nih.gov/19484601/> |
| FACT/GOG-Ntx | English, Chinese | Cancer | internal consistency, construct validity, responsiveness | <https://pubmed.ncbi.nlm.nih.gov/32703223/> |
| FACT-N | English, Chinese | Chemotherapy-induced Neutropenia | construct validity^m^, internal consistency | <https://doi.org/10.1016/j.jpainsymman.2016.03.016> |
| PRO-CTCAE | Chinese | Breast and Colorectal Cancer | Content validity | <https://doi.org/10.1186/s12885-020-07631-5> |
| FoP-Q-SF | English, Chinese | Cancer | test-retest reliability, internal consistency, criterion validity | <https://doi.org/10.1186/s40359-020-0374-0> |
| **Paediatrics** |  |  |  |  |
| KINDL-Kid (Singapore) and KINDL-Kiddo (Singapore) | English | DM | internal consistency, construct validity | <https://hqlo.biomedcentral.com/articles/10.1186/1477-7525-5-4>   <https://link.springer.com/article/10.1007/s11136-004-2957-2> |
| CAQ-B | English | Asthma | content validity, Internal consistency | <https://pubmed.ncbi.nlm.nih.gov/16761907/> |
| PAQLQ | English | Asthma | internal consistency, measurement error, construct validity, responsiveness | <https://apjai-journal.org/wp-content/uploads/2018/02/5PediatricAsthmaQualityofLifeQuestionnaireAPJAIVol17No3Sep1999P155.pdf> |
| ACDS | English | Depression | content validity, construct validity, criterion validity, internal consistency | <https://doi.org/10.1007/s11013-007-9048-0> |
| AADS | English | Depression | content validity, construct validity, internal consistency, inter-rater reliability | <https://pubmed.ncbi.nlm.nih.gov/15561310/> |
| PHQ-A | English | Depressive Disorders | internal consistency, construct validity | <https://doi.org/10.1016/j.psychres.2025.116487> |
| **Kidney Disease** |  |  |  |  |
| KDQOL-SF/KDQOL-36/KDQOL-CF | English, Chinese, Malay | ESRD/Haemodialysis | internal consistency, criterion validity, construct validity, responsiveness | <https://pubmed.ncbi.nlm.nih.gov/22795902/>  <https://link.springer.com/article/10.1007/s40271-013-0015-2>   <https://doi.org/10.1186/1471-2369-11-36>  <https://doi.org/10.1186/s12882-024-03848-9>   <https://doi.org/10.1016/j.jpainsymman.2011.12.282> |
| **Dermatological diseases** |  |  |  |  |
| DSC | English | Dermatology | construct validity, content validity | <https://pubmed.ncbi.nlm.nih.gov/38594911/> |
| RECAP | English | Atopic Eczema | construct validity, internal consistency, measurement error | <https://pubmed.ncbi.nlm.nih.gov/38738773/> |
| SLEQOL/SLEQOL-C | English, Chinese | SLE | internal consistency, test-retest reliability, content validity, responsiveness | <https://doi.org/10.1093/rheumatology/keh605>  <https://doi.org/10.1002/art.22911> |
| HFS-30 | English | Hemifacial Spasm | internal consistency, test-retest reliability, content validity, criterion validity, construct validity, responsiveness | <https://pubmed.ncbi.nlm.nih.gov/15050451/> |
| **Thyroid diseases** |  |  |  |  |
| ThyPRO questionnaire | English | Thyroid/Graves' Disease | internal consistency, content validity, construct validity, responsiveness | <https://pubmed.ncbi.nlm.nih.gov/34236563/> |
| STED-QoL | English | Thyroid Eye Disease | criterion validity | <https://doi.org/10.1167/tvst.7.5.14> |
| **Liver diseases** |  |  |  |  |
| CLDQ/CLDQ-SG | Chinese | Chronic Liver Disease | internal consistency, test-retest reliability, construct validity | <https://journals.sagepub.com/doi/abs/10.1177/2010105818782710>  <https://doi.org/10.1002/jgh3.12239> |
| HQLQ | English | Hepatitis B | internal consistency, test-retest reliability, construct validity | <https://doi.org/10.1111/j.1365-2893.2009.01073.x> |
| **Parkinson's disease** |  |  |  |  |
| PDQ-8 | English, Chinese | Parkinson's Disease | internal consistency, test-retest reliability, construct validity | <https://pubmed.ncbi.nlm.nih.gov/17337275/>  <https://www.sciencedirect.com/science/article/pii/S1353802004000999> |
| PDQ-39 | English, Chinese | Parkinson's Disease | internal consistency, test-retest reliability, content validity, construct validity | <https://pubmed.ncbi.nlm.nih.gov/15789961/>  <https://www.sciencedirect.com/science/article/pii/S1353802004000999> |
| **Gastrointestinal diseases** |  |  |  |  |
| FACT-Ga | English, Chinese | Gastric Cancer | construct validity, internal consistency | <https://doi.org/10.1186/1477-7525-10-145> |
| S-HAQ | English, Chinese | Systemic Sclerosis | Test-retest reliability, construct validity | <https://doi.org/10.1111/j.1756-185x.2012.01731.x> |
| SSc-QoL | English, Chinese | Systemic Sclerosis | Test-retest reliability, construct validity | <https://doi.org/10.1111/j.1756-185x.2012.01731.x> |
| GIT 2.0 | English, Chinese | Systemic Sclerosis | construct validity, test-retest reliability, internal consistency | <https://doi.org/10.1007/s10067-016-3529-x> |
| **Musculoskeletal diseases** |  |  |  |  |
| EFAS | English | Hallux Valgus | internal consistency, construct validity, interpretability | <https://pubmed.ncbi.nlm.nih.gov/36089493/> |
| ASAS HI | English | Axial Spondyloarthritis | Construct validity, content validity, internal consistency, test-retest reliability, measurement error, interpretability | <https://onlinelibrary.wiley.com/doi/abs/10.1111/1756-185X.13640> |
| RAID | English | Rheumatoid Arthritis | content validity, construct validity, internal consistency, test-retest reliability | <https://doi.org/10.1007/s10067-016-3522-4> |
| BASDAI | English | Axial Spondyloarthritis | Internal consistency, Construct validity | <https://pubmed.ncbi.nlm.nih.gov/31721427/> |
| PsAQoL | English, Chinese | Psoriatic Arthritis | internal consistency, Test–retest reliability, content validity, construct validity | <https://bmcmusculoskeletdisord.biomedcentral.com/articles/10.1186/s12891-016-1292-4> |
| ASDAS-CRP | English | Axial Spondyloarthritis | Internal consistency, Construct validity | <https://pubmed.ncbi.nlm.nih.gov/31721427/> |
| GIS | English | Gout | construct validity, internal consistency | <https://doi.org/10.1111/1756-185X.13595> |
| ASQoL | English, Chinese | Ankylosing Spondylitis | internal consistency, test-retest reliability, construct validity, content validity | <https://doi.org/10.1186/s12891-017-1715-x> |
| RAI | English, Chinese | SLE | internal consistency, test-retest reliability, construct validity | <https://pubmed.ncbi.nlm.nih.gov/10405938/>   <https://doi.org/10.1191/0961203302lu156oa> |
| AOFAS | English | Hallux Valgus | construct validity, internal consistency, responsiveness | <https://doi.org/10.1177/19386400221079490> |
| C-MFPDI | Chinese | Inflammatory Arthritis | internal consistency, criterion validity, content validity, test-retest reliability | <https://doi.org/10.1186/s13047-019-0316-3> |
| screening questionnaires for identification of symptomatic KOA | English, Chinese | KOA | content validity, criterion validity, construct validity | <https://doi.org/10.1111/1756-185X.13252> |
| OSIS | English | Arthroscopic Bankart Repair for Shoulder Instability | interpretability | <https://doi.org/10.1177/23259671211060023> |
| **Visual impairment** |  |  |  |  |
| IVI | English, Chinese | Visual Impairment | criterion validity, internal consistency, measurement error | <https://doi.org/10.1007/s11136-015-1141-1> |
| VF-11 | English, Malay | Visual Impairment | internal consistency, criterion validity, content validity, construct validity | <https://doi.org/10.1167/iovs.08-2359> |
| VF-14 | English, Chinese | Cataract Surgery | construct validity, responsiveness | <https://pubmed.ncbi.nlm.nih.gov/23851304/> |
| **Hypertension** |  |  |  |  |
| HTN-SCP | Chinese, Malay | Hypertension | internal consistency, test-retest reliability | [https://doi.org/10.1097/md.0000000000008568](https://doi-org.libproxy1.nus.edu.sg/10.1097/md.0000000000008568) [https://doi.org/10.1136/bmjopen-2017-016152](https://doi-org.libproxy1.nus.edu.sg/10.1136/bmjopen-2017-016152) |
| **AF** |  |  |  |  |
| AF knowledge, attitude and perceptions questionnaire | English, Chinese | AF | internal consistency, content validity, construct validity | <https://doi.org/10.1136/heartasia-2018-011143> |
| **Mental Health** |  |  |  |  |
| SMWEB | English, Chinese, Malay, Tamil | Mental Health | internal consistency, construct validity | <http://dx.doi.org/10.4236/psych.2013.47085> |
| SMPMHI | English | Mental Health | internal consistency, criterion validity, test–retest reliability | <https://pubmed.ncbi.nlm.nih.gov/24307210/> |
| R-PMHI | English | Mental Health | construct validity, criterion validity, Internal consistency | <https://bmcpublichealth.biomedcentral.com/articles/10.1186/s12889-020-08569-w> |
| PMHI | English, Chinese, Malay, Tamil | Mental Health, Schizophrenia, Depression or Anxiety Spectrum Disorder | Internal consistency, criterion validity, construct validity, interpretability | <https://doi.org/10.1186/s12874-018-0487-9>   <https://doi.org/10.1186/s12955-016-0424-8>   <https://doi.org/10.1177/1363461520976045>   <https://doi.org/10.1186/1477-7525-9-92> |
| ReQoL-10 scale | English | Psychosis | Internal consistency, construct validity | <https://doi.org/10.1111/eip.13050> |
| MHC-SF | English | Mental Health | construct validity, internal consistency, criterion validity | <https://doi.org/10.1371/journal.pone.0268232> |
| SQLS | English, Chinese | Schizophrenia | responsiveness, internal consistency, construct validity, test-retest reliability | <https://doi.org/10.1007/s11136-007-9278-1> |
| SWEMWBS | English | Schizophrenia, Depression and Anxiety Spectrum Disorders | construct validity, internal consistency | <https://doi.org/10.1186/s12955-017-0728-3> |
| **Neurological diseases** |  |  |  |  |
| MusiQoL | English | Multiple Sclerosis | internal consistency, content validity, construct validity | <http://www.annals.edu.sg/pdf/40VolNo2Feb2011/V40N2p67.pdf> |

Abbreviations : shortened version of the Western Ontario and McMaster Universities Osteoarthritis Index function scale (Modified ShortMAC-F), Western Ontario and McMaster Universities Osteoarthritis Index (WOMAC), Knee injury and Osteoarthritis Outcome Score (KOOS), Stroke and Aphasia Quality of Life Scale (SAQOL-39 g) and its Mandarin adaptation (SAQOL-CSg), Functional Assessment of Cancer Therapy-Breast (FACT-B), Integrated Palliative care Outcome Scale (IPOS), Problem Areas in Diabetes (PAID), Chinese version of Problem Areas In Diabetes Scale (Sg-Paid-C), Diabetes Health Profile-18 (DHP-18), Diabetic Retinopathy Knowledge and Attitudes (DRKA), Diabetes-Related Nutrition Knowledge (DRNK), Audit of Diabetes-Dependent Quality-of-Life (ADDQoL), Hypoglycemia Fear Survey-II (HFS-II), Singapore Caregiver Quality of Life Scale - 10-item / 15-item (SCQOLS-10/SCQOLS-15), Singapore Caregiver Quality of Life Scale - Dementia (SCQOLS-D), Family Satisfaction with End-of-Life Care (10-Item FAMCARE scale), Quality of Life (QoL), five-level EuroQoL Group's five-dimension questionnaire (EQ-5D-5L/EQ-5D), five-level EuroQoL Group's five-dimension questionnaire descriptive system (EQ-5D-Y DS), 36-Item Short Form Health Survey / 36-Item Short Form Health Survey version 2 (SF-36/SF-36v2), Health Utilities Index Mark 3 (HUI3), Pain Catastrophizing Scale (PCS), World Health Organization Quality of Life-BREF (WHOQOL-BREF), Health-Related Quality of Life (HRQoL), Beck Anxiety Inventory (BAI), Hospital Anxiety and Depression Scales (HADS), Distress Thermometer (DT), Lower Extremity Functional Scale (LEFS), Morisky Medication Adherence Scale (MMAS), Health Assessment Questionnaire (HAQ), Scleroderma Health Assessment Questionnaire (S-HAQ), Health Assessment Questionnaire-Disability Index (HAQ-DI), Oral Impacts on Daily Performances (OIDP), Numerical Rating Scale (NRS), Visual Analogue Scale (VAS), Verbal Rating Scale (VRS), Faces Pain Scale-Revised (FPS-R), Quick Version of the Functional Living Index-Cancer (Quick-FLIC), MD Anderson Dysphagia Inventory (MDADI), Connor-Davidson Resilience Scale (CD-RISC10), Singapore Mental Wellbeing (SMWEB), Medical Outcomes Study Family and Marital Functioning Measures (FMM and MFM), nine-item Patient Health Questionnaire (PHQ-9), one-item Patient Health Questionnaire (PHQ-1), two-item Patient Health Questionnaire (PHQ-2), Adult Sedentary Behaviour Questionnaire (ASBQ), 16-item Quick Inventory of Depressive Symptomatology – Self-Report (QIDS-SR16), Multidimensional Fatigue Syndrome Inventory- Short Form (MFSI-SF), Short Form 6-Dimension (SF-6D), Work Productivity and Activity Impairment (WPAI), European Organization for Research and Treatment of Cancer Quality of Life Questionnaire (EORTC QLQ-C30), Quality of Life Questionnaire – Breast Cancer Specific Module (QLQ-BR23), Short Form-12 version 2 (SF-12v2), Emotion Thermometer (ET), Instrumental Activities of Daily Living (IADL), Center for Epidemiologic Studies Depression Scale (CES-D), 8-item Altarum Consumer Engagement Measure™ (ACE Measure), Rapid Positive Mental Health Instrument (R-PMHI), Control, Autonomy, Self-realization, Pleasure Quality of Life scale (CASP‑11‑SG scale), Functional Assessment of Cancer Therapy - General (FACT-G), Functional Assessment of Cancer Therapy: Cognitive Function (FACT-Cog), Childhood Asthma Questionnaire (CAQ-B), Paediatric Asthma Quality of Life Questionnaire (PAQLQ), Kidney Disease Quality of Life Short Form (KDQOL-SF/KDQOL-36), Kidney Disease Quality of Life Cognitive Function subscale (KDQOL-CF), Dermatology Social Comparison (DSC), Recap of Atopic Eczema Patient-Reported Outcomes (RECAP), Systemic Lupus Erythematosus-Specific Quality-Of-Life instrument (SLEQOL), Systemic Lupus Erythematosus Quality of Life Questionnaire - Chinese version (SLEQOL-C), Hemifacial Spasm-30 (HFS-30), Thyroid-specific quality of life questionnaire (ThyPRO questionnaire), Chronic Liver Disease Questionnaire (CLDQ), Chronic Liver Disease Questionnaire - Singapore-Mandarin version (CLDQ-SG), Hepatitis Quality of Life Questionnaire (HQLQ), 8-item Parkinson's Disease Questionnaire (PDQ-8), 39-item Parkinson's Disease Questionnaire (PDQ-39), Functional Assessment of Cancer Therapy-Gastric Module (FACT-Ga), European Foot and Ankle Society (EFAS), Assessment of Spondyloarthritis International Society Health Index (ASAS HI), Rheumatoid Arthritis Impact of Disease (RAID), Bath Ankylosing Spondylitis Disease Activity Index (BASDAI), Psoriatic Arthritis Quality of Life (PsAQoL), Gout Impact Scale (GIS), Ankylosing Spondylitis quality of life (ASQoL), Rheumatology Attitudes Index (RAI), American Orthopaedic Foot and Ankle Society (AOFAS), Chinese Manchester foot pain and disability index (C-MFPDI), Knee Osteoarthritis (KOA), Osteoarthritis (OA), Impact of Vision Impairment (IVI), Visual Function Index-11 (VF-11), Hypertension Self-Care Profile (HTN-SCP), Atrial fibrillation (AF), Singapore (SG), Diabetes mellitus (DM), Hypertension (HTN), Total Knee Replacement /Total Knee Arthroplasty (TKR/TKA), Value Driven Care (VDC), Systemic lupus erythematosus (SLE), Age-Related Macular Degeneration (ARMD), short multidimensional positive mental health instrument (SMPMHI), End stage renal disease (ESRD), Oxford Knee Score (OKS), Short-Form of the McGill Pain Questionnaire (MPQ), positive mental health instrument (PMHI), Global Physical Activity Questionnaire (GPAQ), Ankylosing Spondylitis Disease Activity Score with C-reactive protein (ASDAS-CRP), Oxford Hip Score (OHS), Oxford Shoulder Instability Score (OSIS), Asian Children Depression Scale (ACDS), Asian Adolescent Depression Scale (AADS), Geriatric Depression Screening Scale (GDS-15), Minimal clinically important difference (MCID), Visual Function Index-14 (VF-14), Kansas City Cardiomyopathy Questionnaire (KCCQ), Total Hip Arthroplasty (THA), Arthroscopic Rotator Cuff Repair (Arthroscopic RCR), Functional Assessment of Cancer Therapy/Gynecologic Oncology Group—Neurotoxicity (FACT/GOG-Ntx), U.S. National Cancer Institute’s Patient-Reported Outcomes version of the Common Terminology Criteria for Adverse Events (PRO-CTCAE), Recovering Quality of Life 10-item (ReQoL-10) scale, Systemic Sclerosis Quality of Life scale (SSc-QoL), 13-items World Health Organization Quality of Life Assessment-Older Adults Module (WHOQOL-AGE), Mental Health Continuum-Short Form (MHC-SF), Fear of Progression Questionnaire – Short Form (FoP-Q-SF), Schizophrenia Quality of Life Scale (SQLS), Acceptance of Chronic Health Conditions (ACHC), Functional Assessment of Cancer Therapy-Neutropenia (FACT-N), Short Warwick Edinburgh Mental Well-Being Scale (SWEMWBS), Patient Health Questionnaire for Adolescents (PHQ-A), Gastrointestinal Tract Instrument (GIT), Autism Spectrum Disorder (ASD), Multiple sclerosis international quality of life questionnaire (MusiQoL), Singapore Thyroid Eye Disease Quality of Life questionnaire (STED-QoL), measurement equivalence/invariance classified as construct validity (construct validity^m^)
